# Supplementary material for: Health data ecosystem in Pakistan: a multisectoral qualitative assessment of needs and opportunities
Source: BMJ Open. 2023 Sep 21;13(9):e071616. doi: 10.1136/bmjopen-2023-071616 (PMC10514666; doi:10.1136/bmjopen-2023-071616)
Supplement: Supplementary data [file bmjopen-2023-071616supp001.pdf]

Semi-structured interview guide

Section 1 : Understanding the health data landscape for Pakistan

What type of health data exists in Pakistan?

Potential prompts in case of a brief reply

- What type of data at a national/regional/global level supports your decision-making ability/research work?
- What type of health data would further support your ability to make informed decisions?
- Is health data at a Pakistan level accessible?
- Is health data at a Pakistan level of good quality? (define quality)

Section 2: Understanding the application of a gender and equity lens to data

Potential prompts in case of a brief reply (mostly guided by the interviewee's response)

- Do we know how to apply a gender/equity lens to our data (disaggregation, analysis etc)
- What population group do you not frequently see available data about?

Section 3: Understanding the organizational handle on health data and its current role

Potential prompts in case of a brief reply (mostly guided by the interviewee's response)

- What health data does your organization hold and to what level does your organization engage with the data for decision making.
- How equipped are organizations to manage the health data they hold?
- What kind of infrastructure/software does your organization have? Is it sufficient?

Section 4: Understanding perceptions around developing a health data science training program/curriculum

How effective do you think the introduction of a health data science training curriculum will be, to address barriers?

Potential prompts in case of a brief reply (mostly guided by the interviewee's response)

- What type of training in data science would be most beneficial to you and why?
- What health data science curriculum/training programs exist and are useful?
- Do you think there's an existing need for development of such a program in Pakistan? Why or why not?
- What barriers should such a training program address?
- What components should the health data science training curriculum have?
