# Supplementary material for: Health data ecosystem in Pakistan: a multisectoral qualitative assessment of needs and opportunities
Source: BMJ Open. 2023 Sep 21;13(9):e071616. doi: 10.1136/bmjopen-2023-071616 (PMC10514666; doi:10.1136/bmjopen-2023-071616)
Supplement: Author's manuscript [file bmjopen-2023-071616.draft_revisions.pdf]

BMJ Open is committed to open peer review. As part of this commitment we make the peer review history of every article we publish publicly available.

When an article is published we post the peer reviewers' comments and the authors' responses online. We also post the versions of the paper that were used during peer review. These are the versions that the peer review comments apply to.

The versions of the paper that follow are the versions that were submitted during the peer review process. They are not the versions of record or the final published versions. They should not be cited or distributed as the published version of this manuscript.

BMJ Open is an open access journal and the full, final, typeset and author-corrected version of record of the manuscript is available on our site with no access controls, subscription charges or pay-per-view fees (http://bmjopen.bmj.com).

If you have any questions on BMJ Open's open peer review process please email info.bmjopen@bmj.com

BMJ Open

Health Data Ecosystem in Pakistan – a multi-sectoral qualitative assessment of needs and opportunities

Journal:	*BMJ Open*
Manuscript ID	bmjopen-2023-071616
Article Type:	Original research
Date Submitted by the Author:	09-Jan-2023
Complete List of Authors:	Mahmood, Sana; Aga Khan University; Aga Khan University, Department of Community Health Sciences<br>Noorali, Ali Aahil; The Aga Khan University<br>Manji, Afshan; Aga Khan University, Department of Medicine; Aga Khan University<br>Abbas, Saadia; Aga Khan University, Department of Medicine<br>Qamar, Javeria Bilal; Aga Khan University, Department of Medicine<br>Afzal, Noreen; The Aga Khan University<br>Siddiqi, Sameen; Aga Khan University Medical College Pakistan, Community Health Sciences Department<br>Hoodbhoy, Zahra; Aga Khan University, Department of Pediatrics and Child Health<br>Virani, Salim; Michael E. DeBakey Veterans Affairs Medical Center HSR&D, Health Policy, Quality & Informatics Program,<br>Bhutta, Zulfiqar; Hosp Sick Children, Centre for Global Child Health<br>Samad, Zainab; Aga Khan University; Duke Clinical Research Institute, Department of Medicine
Keywords:	QUALITATIVE RESEARCH, PUBLIC HEALTH, Quality in health care < HEALTH SERVICES ADMINISTRATION & MANAGEMENT

SCHOLARONE™
Manuscripts

I, the Submitting Author has the right to grant and does grant on behalf of all authors of the Work (as defined in the below author licence), an exclusive licence and/or a non-exclusive licence for contributions from authors who are: i) UK Crown employees; ii) where BMJ has agreed a CC-BY licence shall apply, and/or iii) in accordance with the terms applicable for US Federal Government officers or employees acting as part of their official duties; on a worldwide, perpetual, irrevocable, royalty-free basis to BMJ Publishing Group Ltd ("BMJ") its licensees and where the relevant Journal is co-owned by BMJ to the co-owners of the Journal, to publish the Work in this journal and any other BMJ products and to exploit all rights, as set out in our licence.

The Submitting Author accepts and understands that any supply made under these terms is made by BMJ to the Submitting Author unless you are acting as an employee on behalf of your employer or a postgraduate student of an affiliated institution which is paying any applicable article publishing charge ("APC") for Open Access articles. Where the Submitting Author wishes to make the Work available on an Open Access basis (and intends to pay the relevant APC), the terms of reuse of such Open Access shall be governed by a Creative Commons licence – details of these licences and which Creative Commons licence will apply to this Work are set out in our licence referred to above.

Other than as permitted in any relevant BMJ Author's Self Archiving Policies, I confirm this Work has not been accepted for publication elsewhere, is not being considered for publication elsewhere and does not duplicate material already published. I confirm all authors consent to publication of this Work and authorise the granting of this licence.

For peer review only

Health Data Ecosystem in Pakistan – a multi-sectoral qualitative assessment of needs and opportunities

Authors: Sana Mahmood EdM [1,2,3], Ali Aahil Noorali MBBS [4,5], Afshan Manji MSc [4,5], Saadia Abbas [4], Javeria Bilal Qamar [4], Noreen Afzal MPhil [1], Sameen Siddiqi DrMed [2], Zahra Hoodbhoy PhD[6], Salim S. Virani PhD[7], Zulfiqar A. Bhutta, PhD [3], Zainab Samad MHS [3,4,8]

Affiliations:

[1] Dean's Office, Medical College, Aga Khan University, Karachi, Pakistan

[2] Department of Community Health Sciences, Aga Khan University, Karachi, Pakistan

[3] Institute of Global Health and Development, Aga Khan University, Karachi, Pakistan

[4] Department of Medicine, Medical College, Aga Khan University, Karachi, Pakistan

[5] CITRIC Health Data Science Center, Medical College, Aga Khan University, Karachi, Pakistan

[6] Department of Pediatrics and Child Health, Aga Khan University, Karachi, Pakistan

[7] Division of Cardiology, Department of Medicine, Baylor College of Medicine, USA

[8] Division of Cardiology, Department of Medicine, Duke University, Duke Global Health Institute, Duke Clinical Research Institute, Durham, NC

Corresponding Author:

Zainab Samad, MBBS, MHS

Aga Khan University, Stadium Road, Karachi, Pakistan, 74800

Email: samad.zainab@aku.edu

Phone: 03002017196

For peer review only - http://bmjopen.bmj.com/site/about/guidelines.xhtml

Word count:

3508

For peer review only

For peer review only - http://bmjopen.bmj.com/site/about/guidelines.xhtml

Abstract

Objective:

Data are essential for tracking and monitoring of progress on health-related sustainable development goals (SDGs). But the capacity to analyze subnational and granular data is limited in low and middle-income countries. Through an exploratory qualitative approach, we aimed to understand the current landscape and perceptions around the health data ecosystem among key stakeholders in the healthcare and affiliated systems.

Design:

An exploratory qualitative study design was employed.

Setting:

This study was conducted at the Aga Khan University, Karachi, Pakistan.

Participants:

We conducted semi-structured, in-depth interviews with multidisciplinary and multisectoral stakeholders from academia, hospital management, government, NGOs, and private entities till thematic saturation was achieved. Interviews were recorded and transcribed, followed by thematic analysis using NVivo.

Results:

Thematic analysis of 15 in-depth interviews revealed five major themes: institutions are collecting data, but face barriers such as lack of structured data, data reliability and limited analytical ability for effective utilization of data; there is openness and enthusiasm for sharing data for advancing health; barriers to data sharing including accuracy, privacy and interoperability of data; gender information and health equity is not necessarily captured

For peer review only - http://bmjopen.bmj.com/site/about/guidelines.xhtml

routinely or deliberately ; and there is limited capacity in the area of both human capital and infrastructure, for being able to use data to advance health.

Conclusions:

Our study identified key areas of focus that can inform a national health data roadmap and ecosystem in Pakistan.

For peer review only

For peer review only - http://bmjopen.bmj.com/site/about/guidelines.xhtml

Strengths and limitations of this study

- Our study is the first, multi-disciplinary endeavor to understand perceptions on health data and health data science in Pakistan.
- We present perspectives from a low resource setting which has contextual relevancy and implications for other LMICs in the region.
- Our findings might not be generalizable to high income countries.

For peer review only

Introduction

Data are essential for tracking and monitoring of progress on health-related sustainable development goals (SDGs). (1–3) While data and data analytics are being used in high income countries (HICs) to track human wellbeing, improve health equity, health outcomes, and continuously inform healthcare systems, their use in low middle income countries (LMICs) is lagging.(4–8) Several LMICs are trailing in their progress towards achievement of SDGs; leveraging data and data science methods could represent an important cost-effective opportunity for monitoring progress on health-related SDGs. (1,9–11)

With a 220 million population, Pakistan, the fifth most populous LMIC, has a high mortality and morbidity burden for various diseases; however, its health system and health information system capacity is nascent. (12,13) For example, a recent WHO survey report highlighted that only 40 % of births in Pakistan are registered. (14) Management of health systems and improving health outcomes in a large country like Pakistan will require organization and use of available health data and potentially novel digital data exhausts. (15)

During the COVID-19 pandemic, data were made nationally available in almost real-time, and data science methods were used to inform health policy and population level interventions such as smart lock downs and vaccinations efforts. (16,17) Multistakeholder and interprovincial collaboration underpinned this successful effort and highlighted that a national health data ecosystem should be developed outside of crisis situations.

To inform future efforts, an understanding of current perceptions and a mapping of national landscape is required on health data and health data science methods. To this end, we adopted a qualitative approach to understand the current landscape and perceptions on data in decision-making and health policy among a wide range of stakeholders.

For peer review only - http://bmjopen.bmj.com/site/about/guidelines.xhtml

Methods

Study Design and Setting:

This was an exploratory qualitative study with the primary objectives to comprehend the scope of health data science in Pakistan, the knowledge, and attitudes around developing partnerships and sharing data, and perceptions around the need for developing health data science capacity in Pakistan.

The study was led by investigators at the Aga Khan University (AKU), Karachi, Pakistan. With a forty-year presence in Pakistan, AKU has well established partnerships at both provincial and national levels, with government and academia, enabling regular engagement in interdisciplinary policy discussions and fora.

Study instrument:

A semi-structured interview guide was designed using carefully curated questions (available in supplement 1). The guide prompted a detailed discussion on the landscape and scope of existing health data. Further discussion was rooted in potential facilitators and barriers to building a national health data collaborative that would contribute to improved health outcomes in Pakistan. This included understanding the nature of existing policies and collaboratives, availability and need of human capital for health data initiatives, and structures—from governance to infrastructure, which were present or would need to be developed and implemented to allow for organizations across sectors to comfortably share data to advance health outcomes in Pakistan. The guide was pilot tested among a diverse cohort of four individuals and judged for clarity of questions as well as face and content validity. Feedback from the pilot testing was incorporated to address gaps in the interview guide.

For peer review only - http://bmjopen.bmj.com/site/about/guidelines.xhtml

Data collection

Interviews were conducted by two female investigators; ZS (MHS) and SM (Ed.M.), while research staff (AAN, AA, SA, JBQ) acted as observers. Standardization was maintained across all interviews by ensuring that the same two interviewers conducted all the interviews with the same guide. Both interviewers had prior experience of conducting qualitative interviews. Participants were interviewed once. Each interview was conducted online for a duration that varied between 30 minutes and 2 hours.

Sampling, Inclusion and Exclusion Criteria:

A scoping exercise was conducted to identify experts and relevant institutions. Through discussion, the investigators collectively identified key sectors in the health ecosystem of Pakistan for a landscape analysis which formed the inclusion criteria: 1. University and academia with faculty in health and/or information technology (IT), 2. Senior level hospital management (both private and public) 3. Government ministers (federal and provincial/state), 4. Non-governmental organizations (NGOs) and 5. Private-sector organizations (pharmaceuticals, finance). (Figure 1) There were no major or minor exclusion criteria.

Following convenience sampling to select key stakeholders, invites were forwarded via email, and interviews arranged in accordance with mutual availability. Thematic saturation was reached at 15 which comprised the final study sample.

Data Analysis:

Grounded theory and the six-step method of thematic analysis of Braun and Clarke (2006) guided the analytical process. (18) Interviews were first audio recorded and transcribed verbatim.

For peer review only - http://bmjopen.bmj.com/site/about/guidelines.xhtml

Since the research staff were not only observers, but also transcribers for the interviews, these roles helped them get familiarized and immersed with the collected data. Transcripts were imported into NVivo (version 12) and read line by line to inductively code the data. An initial code book was developed by AAN and AM, which was refined through iteration and consensus among research staff. This framework helped in standardization of codes applied in all transcripts.

Data coded under the framework were then grouped together based on similarities to identify major themes, which were paired with direct verbatim quotations from the interviewees. The themes were then reviewed to ensure adequate data and participant quotations supported the creation of each theme. All themes were then defined to convey an adequate description of its subthemes and relevant data. Lastly, the results were written in a format to describe the analyzed data.

Patient and Public Involvement

Patients and public were not involved in the research design, analysis and dissemination of the findings.

Ethical Considerations:

The study received approval from the Ethical Review Committee at AKU (ERC # 2021-5839-16883). Written informed consent over email for the study was obtained from each participant before starting the interview.

For peer review only

For peer review only - http://bmjopen.bmj.com/site/about/guidelines.xhtml

Results

We conducted 15 in-depth interviews with a diverse range of stakeholders from five centralized cohorts. Demographic characteristics of the participants are described in table 1. (Figure 1). Thematic analysis generated five overarching themes: 1. Institutions are collecting data, but face barriers of unstructured data, data reliability and limited analytical ability for effective utilization of data; 2. There is openness and enthusiasm for sharing data for advancing health; 3. Barriers to data sharing including accuracy, privacy and interoperability of data; 4. Gender information and health equity is not necessarily captured routinely or deliberately and 5. There is limited capacity in the area of both human capital and infrastructure, for being able to use data to advance health. (Figure 2)

Table 1: Demographic characteristics of study participants (n=15)

**Sector**	**n(%)**
Academia	1
Hospitals	2
Government	3
Non-governmental organizations (NGOs)	3
Private-sector organizations	6
**Designation**	
Mid-level management	5
Chief medical officer	2
Health minister	2
Senior management	6

For peer review only - http://bmjopen.bmj.com/site/about/guidelines.xhtml

Theme 1: Institutions are collecting data, but face barriers such as lack of structured data, data reliability and limited analytical ability for effective utilization of data

Experts communicated that there are several initiatives at the intersection of health and data, but these exist in silos. Institutions have large volumes of operational data, but these datasets lack structure and coherence, which impedes the ability to gauge actionable insights from it. Most participants shared the perception of a lack of an appropriately designed system to help collate data to their most desired format. Many interviewees felt that where data did exist, there were other obstacles like significant deficits in staffing and the inability to utilize the collected data. The officials within the ministry of health stated that *"We find that getting population level information is really difficult. And you can get this information, but it's not really formalized - you just hear verbal estimates. So, in terms of planning, there is no common information base that people have that is like the gold standard."*

This sentiment was shared by another expert in epidemiology at a tertiary care hospital, who noted that: *"The reality was when I came in, I realized that the structure of the data being collected, was not conducive to be pulled out, right, so we couldn't do any research."*

Some participants reported that sharing and dissemination of data with external stakeholders is not always prioritized by many organizations. Although data are being rigorously collected, there is a lack of clarity on how to share data and in what structure, which appears to be a bigger barrier than missingness of data. An expert in digital health strategy noted *"I would think that the dissemination of the data is a far bigger issue than the data holes being there. So, if you start digging, you actually find data sets, but you see that no one is aware of them, even though a lot of activity has happened"*. The structure of available data was cited as another barrier because of a lack of electronic systems.

For peer review only - http://bmjopen.bmj.com/site/about/guidelines.xhtml

Experts shared that even if the data exists, dissemination of data is always an issue because data governance is a nascent field in Pakistan. The term governance is also used broadly by interviewees that use it to refer to privacy and security of data as well as an overarching governing structure to establish appropriate ownership of data. A premier bank entity leader noted that this also holds true for other sectors of Pakistan, such as the well-resourced finance sector, where one of the country's major banks is still working through the early stages of data governance.

Theme 2: There is openness and enthusiasm for sharing data for advancing health

In light of a shared vision to improve health outcomes in Pakistan, leaders indicated overall willingness to share data and partner for this common mission, expressing keen interest and stating their openness to proposals and collaborations. A government leader observed: *"I think we are open to proposals where, say, we make data in specific areas available.'"*

Interestingly, an expert shared insights about ensuring that a shared collaborative keeps 'democracy of data' as a central guiding principle. An epidemiologist at a tertiary care hospital noted *"I think there has to be democracy of data sharing within an organization because there's no point hanging on to data, and not sharing it so that somebody can make use of it, and that is one of the problems; people hang on to data as a good treasure that they cannot share with anybody."*

Theme 3: Barriers to data sharing including accuracy, privacy and interoperability of data

Most experts shared that certain challenges and pertinent questions would need to be accounted for to build a sustainable, shared, accessible data ecosystem. These include the validity and

For peer review only - http://bmjopen.bmj.com/site/about/guidelines.xhtml

accuracy of datasets themselves, privacy and regulatory framework around data sharing, addressing systemic differences between different sectors and inadequate workforce and training. A manager and planning executive at a bank, noted: *"Some concerns in data sharing include who will own the data, what will be done with the data? Will the data remain valid or not, will transparency be maintained, privacy rules will be followed or not, ownership of the data? Where will data be used ultimately."*

Similarly, a chief medical officer at a tertiary hospital mentioned that: *"There needs to be a lot more structure put into data sharing, and by structure, what I mean is that rules and regulations (which need to be set up a priori). That will really give confidence to individual institutions and individuals who own that data - that their data is going to be used properly, reliably and honestly".*

Interoperability of data sets was reported as a big challenge due to differences in dataset formats across different institutions. This lack of interoperability is further pronounced when complemented with differences in the approach of private vs public sectors and inpatient vs outpatient data. A leader in healthcare administration stated that *"Record keeping is something that is very poor there. In-patient record keeping is there, but there is nothing for out-patients at all. Private sector does keep the record, but the public sector does not. But the private sector does not share that data at all."*

Theme 4: Gender information and health equity is not necessarily captured routinely or deliberately

Most participants noted that the gender and equity lens have not been widely considered, neither during the collection and analysis of health data, nor in the design of research and data driven

For peer review only - http://bmjopen.bmj.com/site/about/guidelines.xhtml

initiatives. With regards to gender, a leader at a non-profit organization explained that: *"Disaggregated data by gender is probably a large problem nationally, particularly with development indicators."*

Equity is often overlooked in conversations around data, and when considered, the level of commitment is inadequate. A governmental leader mentioned that: *"Unfortunately, I think equity is more a function of conversations with development partners. And it does translate to some commitment, but not the level of commitment that should be the case."*

Theme 5: There is limited capacity in the area of both human capital and infrastructure, for being able to use data to advance health

Participants remarked that data sharing through a collaborative, on its own, would also require capacity-building, which currently presents a barrier to achieving a cohesive data sharing initiative.

A manager at an NGO noted: *"The issue is not whether people are willing to share data. But certain organizations, traditional non-profit ones for example, don't have data teams or data managers (because of cost budgetary constraints). So, I think that these organizations often don't have the capacity to manage data."*

It was also mentioned that lack of capacity building was a barrier to successfully analyze and evaluate data. A provincial healthcare leader noted: *"The main problem here is that we don't have an HR [human resource] there or a proper computerized system there to log in that data and upload it. We have a lot of restrictions in the IT department."* Similarly, a senior leader at a tertiary care hospital mentioned: "*We have the data, but we don't have the capacity and capability to analyze it and make changes in healthcare."*

For peer review only - http://bmjopen.bmj.com/site/about/guidelines.xhtml

A representative from a large public sector tertiary care hospital reported their initiative of data automation and the barriers associated with it:

"We have begun an initiative at our hospital where we are starting to do automation of the data and that is happening … That is also not working because our staffing there has been rejected so we are now trying to have a medical record system as an operative thing to see how the medical reforms will work."

An NGO leader described the process, components, and importance of building a data driven team – a cohesive unit of trained individuals that understands the application of data sciences to health. He stated: "*There are four skills that I think are really important in building out a data team that we found. One is data engineering, which is just someone who can query databases, particularly complex databases...Then, I think the next skill that we found really useful is analytics, which is how to develop dashboards. And that is a very easily trainable skill. So the third skill that's (commonly) not there is knowing what dashboard to make. And the fourth skill is data science, which is basically being able to model data and that's an even rarer skill especially locally."*

For peer review only - http://bmjopen.bmj.com/site/about/guidelines.xhtml

Discussion

Our study is the first, multi-disciplinary endeavor to understand perceptions on health data and health data science in Pakistan. The main finding from our qualitative analysis is that the scope of data science in health for advancing health outcomes, is far-reaching in Pakistan and likely in other LMICs where organizations have collected a great deal of data but are in the early stages of understanding how best to leverage and utilize this data. Furthermore, there is potential for establishing a health data ecosystem comprised of a health data collaborative with an appropriate governance structure, and capacity building initiatives. Finally, gender and equity must be intentionally included in the design of any collaborative and is critical to developing a health data strategy that looks to advance health in Pakistan in an equitable manner.

Even though the far-reaching scope of health data and data science methods in healthcare and their potential benefits are recognized by developing countries, development of a national data collaborative that might serve as a foundational block of a larger health data ecosystem is a complex endeavor and presents some challenges. (11) A similar viewpoint was expressed by the participants in our study, who indicated that even though independent and national efforts are being made to allow for digitization and automation in healthcare, we as a country are at the very nascent stages of using data to progress a national health agenda. A principal lever for this agenda is timely access to the right information, but this has been a scarce resource in LMICs. (19)

Lack of rigorous and structured systems, problems with accuracy, credibility and completeness, inadequacy of trained personnel with core competencies, and unavailability of analytic tools were core obstacles highlighted by experts. Furthermore, there are very limited efforts to

For peer review only - http://bmjopen.bmj.com/site/about/guidelines.xhtml

propagate a multimodal, multisectoral, and multidisciplinary approach to data, leading to a conspicuous lack of a central repository of information in LMICs like Pakistan. (9) In line with recent literature, a key concern highlighted by participants in data sharing was safeguarding their privacy, confidentiality, and security, with all interviewees agreeing to the need of a governance and regulatory framework being set up a priori to ensure data transparency and maintaining the trust of all parties involved that their data will be used honestly and reliably. (9,20)

Our participants also stated that the analysis, design, and collection of health data does not currently support gender and equity lens as the core of any organization. Disaggregation of data by gender is a problem nationally and is difficult to find. Literature suggests that health system policy development does not always pay adequate attention to gender and even when policies do include gender, intentions can evaporate when it comes to actual implementation. (21,22) Tannenbaum et al. and The World Bank note that gender data is a powerful tool for improving lives as lack of disaggregated gender information has resulted in an incomplete disease understanding.(23,24) Furthermore, gender equity is an integral component of social responsibility and according to International Organization for Standardization (ISO) 26000 Sustainable Development Goal 5 (Guidance on Social Responsibility), whereby the standard denotes the importance of having gender-inclusive leadership and governance in ensuring elimination of gender bias and promotion of gender parity. (25)

Our study participants emphasized that translation and evidence synthesis require significant capacity building. Organizations collect and store data, have information management systems, but that data is not being utilized in the most effective way, due to limited capacity and skillsets. A systematic review reflects on the importance of ongoing training and multilevel strategies needed in development of such programs, and how capacity building can influence different

For peer review only - http://bmjopen.bmj.com/site/about/guidelines.xhtml

levels of entire organizations and systems. (26) The types of interventions assessed included internet-based teaching and workshops. The results of a worldwide cross-sectional survey by Kaggle et. al, illustrates the extent to which companies in various countries have adapted to machine learning models, with Israel surpassing even the United States. (27) Moreover, 21% of respondents' companies were exploring machine learning methods with the goal of putting a model into commercial use. A Global System for Mobile Communications report on artificial intelligence use in LMICs discusses the major barriers to big data science adoption for LMICs. (28) These include shortage of knowledge, skills and expertise, unreliable power generation, frequent power outages and fluctuations, lack of access to sufficient computing power, and unavailability or inaccessibility of quality data in LMICs, particularly in fragile or conflict affected contexts.

A national strategy on developing a health data ecosystem and data collaborative for Pakistan necessitates that the gaps identified globally and in our qualitative interviews are bridged and data are put into action. In this regard, a national health digital framework has recently been developed by the Ministry of Health, which can be used for developing a large-scale roadmap and as a key tool for stakeholder engagement and buy in. As noted by the healthcare experts, the roadmap is to help healthcare professionals use data science principles to inform decision making, uplifting research, and guiding clinical approaches to improve healthcare delivery. (29,30)

This study has a few limitations. Our findings might not be generalizable to high income countries. However, we present perspectives from a low resource setting which has contextual relevancy and implications for other LMICs in the region. Qualitative interviews focused on perspectives from key management leads at major institutions. This was primarily because the

For peer review only - http://bmjopen.bmj.com/site/about/guidelines.xhtml

scope and objectives of this exercise were to assimilate input from experts and leaders in management, policy, and healthcare. A next step, on establishing a health data collaborative, will be to ensure data and perspectives from patients and communities, who serve as a key stakeholder in healthcare systems.

Conclusion

This systematic approach to understanding the perceptions around the health data ecosystem in Pakistan highlighted important opportunities and barriers that need to be addressed to further develop a health ecosystem in Pakistan. Creation of appropriate governance, rules and regulations, gender and equity indicators are important principles to consider for planning any national health data collaborative. To enable this ecosystem, collaboration is required on strategic outlining of how data can be collated, organized, curated, updated, and finally pipelined. For achieving this goal, building data science capacity within organizations would be critical, thus providing the ability to leverage health data to its full potential for informed decision making.

For peer review only

For peer review only - http://bmjopen.bmj.com/site/about/guidelines.xhtml

Contributors

All authors confirm that they had full access to all the data in the study and accept responsibility to submit for publication. ZM conceived of, designed the study and acquired funding. ZS and SM collected the data. SA, JQB were involved in data curation. AAN and AM analyzed and interpreted the data. ZM, SM, AAN, AM, and NA wrote the original draft of the manuscript. All authors contributed to reviewing and editing the manuscript.

Patient and Public Involvement

Patients and public were not involved in the research design, analysis and dissemination of the findings.

Declaration of interests

We declare no competing interests.

Funding

This work was supported through a grant from the Bill & Melinda Gates Foundation (grant number: INV-021944).

Data availability statement

No data are available.

For peer review only - http://bmjopen.bmj.com/site/about/guidelines.xhtml

For peer review only

Ethics approval

The study received approval from the Ethical Review Committee at AKU (ERC # 2021-5839-16883).

Licence statement

I, the Submitting Author has the right to grant and does grant on behalf of all authors of the Work (as defined in the below author licence), an exclusive licence and/or a non-exclusive licence for contributions from authors who are: i) UK Crown employees; ii) where BMJ has agreed a CC-BY licence shall apply, and/or iii) in accordance with the terms applicable for US Federal Government officers or employees acting as part of their official duties; on a worldwide, perpetual, irrevocable, royalty-free basis to BMJ Publishing Group Ltd ("BMJ") its licensees and where the relevant Journal is co-owned by BMJ to the co-owners of the Journal, to publish the Work in BMJ Open and any other BMJ products and to exploit all rights, as set out in our licence.

The Submitting Author accepts and understands that any supply made under these terms is made by BMJ to the Submitting Author unless you are acting as an employee on behalf of your employer or a postgraduate student of an affiliated institution which is paying any applicable article publishing charge ("APC") for Open Access articles. Where the Submitting Author wishes to make the Work available on an Open Access basis (and intends to pay the relevant APC), the terms of reuse of such Open Access shall be governed by a Creative Commons licence

For peer review only - http://bmjopen.bmj.com/site/about/guidelines.xhtml

– details of these licences and which Creative Commons licence will apply to this Work are set out in our licence referred to above.

References

1. Measuring progress from 1990 to 2017 and projecting attainment to 2030 of the health-related Sustainable Development Goals for 195 countries and territories: a systematic analysis for the Global Burden of Disease Study 2017. Lancet (London, England). 2018 Nov;392(10159):2091–138.
2. Transforming our world: the 2030 Agenda for Sustainable Development | Department of Economic and Social Affairs [Internet]. [cited 2022 Nov 21]. Available from: https://sdgs.un.org/2030agenda
3. Sachs JD, Schmidt-Traub G, Mazzucato M, Messner D, Nakicenovic N, Rockström J. Six Transformations to achieve the Sustainable Development Goals. Nat Sustain 2019 29 [Internet]. 2019 Aug 26 [cited 2022 Nov 21];2(9):805–14. Available from: https://www.nature.com/articles/s41893-019-0352-9
4. Bhavnani SP, Muñoz D, Bagai A. Data Science in Healthcare: Implications for Early Career Investigators. Circ Cardiovasc Qual Outcomes. 2016 Nov;9(6):683–7.
5. Sharma A, Harrington RA, McClellan MB, Turakhia MP, Eapen ZJ, Steinhubl S, et al. Using Digital Health Technology to Better Generate Evidence and Deliver Evidence-Based Care. J Am Coll Cardiol. 2018 Jun;71(23):2680–90.
6. Ting DSW, Carin L, Dzau V, Wong TY. Digital technology and COVID-19. Nat Med. 2020 Apr;26(4):459–61.
7. Imoto S, Hasegawa T, Yamaguchi R. Data science and precision health care. Nutr Rev. 2020 Dec;78(12 Suppl 2):53–7.

For peer review only - http://bmjopen.bmj.com/site/about/guidelines.xhtml

8. Andreu-Perez J, Poon CCY, Merrifield RD, Wong STC, Yang GZ. Big data for health. IEEE J Biomed Heal informatics. 2015 Jul;19(4):1193–208.

9. Bezuidenhout L, Chakauya E. Hidden concerns of sharing research data by low/middle-income country scientists. Glob Bioeth = Probl di Bioet. 2018;29(1):39–54.

10. Wyber R, Vaillancourt S, Perry W, Mannava P, Folaranmi T, Celi LA. Big data in global health: improving health in low- and middle-income countries. Bull World Health Organ. 2015 Mar;93(3):203–8.

11. Naseem M, Akhund R, Arshad H, Ibrahim MT. Exploring the Potential of Artificial Intelligence and Machine Learning to Combat COVID-19 and Existing Opportunities for LMIC: A Scoping Review. J Prim Care Community Health. 2020;11:2150132720963634.

12. Brief on Census -2017 | Pakistan Bureau of Statistics [Internet]. [cited 2022 Nov 21]. Available from: https://www.pbs.gov.pk/content/brief-census-2017

13. Roth GA, Abate D, Abate KH, Abay SM, Abbafati C, Abbasi N, et al. Global, regional, and national age-sex-specific mortality for 282 causes of death in 195 countries and territories, 1980–2017: a systematic analysis for the Global Burden of Disease Study 2017. Lancet [Internet]. 2018 Nov 10 [cited 2022 Nov 21];392(10159):1736–88. Available from: http://www.thelancet.com/article/S0140673618322037/fulltext

14. Civil registration and vital statistics (CRVS) - WHO [Internet]. [cited 2022 Nov 21]. Available from: https://www.who.int/data/data-collection-tools/civil-registration-and-vital-statistics-(crvs)

15. Samad Z, Mahmood S, Siddiqui S, Bhutta ZA. Imagining a lean and agile digital health ecosystem - a measure of pandemic responsiveness. J Glob Health. 2020 Dec;10(2):20391.

For peer review only - http://bmjopen.bmj.com/site/about/guidelines.xhtml

16. Akhtar H, Afridi M, Akhtar S, Ahmad H, Ali S, Khalid S, et al. Pakistan's Response to COVID-19: Overcoming National and International Hypes to Fight the Pandemic. JMIR Public Heal Surveill [Internet]. 2021 May 1 [cited 2022 Nov 21];7(5). Available from: /pmc/articles/PMC8136406/

17. Haq ZU, Mirza Z, Oyewale TO, Sultan F. Leaving no one behind: Pakistan's risk communication and community engagement during COVID-19. J Glob Health [Internet]. 2021 [cited 2022 Nov 21];11:1–5. Available from: https://pubmed.ncbi.nlm.nih.gov/34386212/

18. Kiger ME, Varpio L. Thematic analysis of qualitative data: AMEE Guide No. 131. Med Teach [Internet]. 2020 Aug 2 [cited 2022 Nov 21];42(8):846–54. Available from: https://pubmed.ncbi.nlm.nih.gov/32356468/

19. Harrison K, Rahimi N, Danovaro-Holliday MC. Factors limiting data quality in the expanded programme on immunization in low and middle-income countries: A scoping review. Vaccine. 2020 Jun;38(30):4652–63.

20. Tiffin N, George A, LeFevre AE. How to use relevant data for maximal benefit with minimal risk: digital health data governance to protect vulnerable populations in low-income and middle-income countries. BMJ Glob Heal. 2019;4(2):e001395.

21. Morgan R, Ayiasi RM, Barman D, Buzuzi S, Ssemugabo C, Ezumah N, et al. Gendered health systems: evidence from low- and middle-income countries. Heal Res policy Syst. 2018 Jul;16(1):58.

22. Theobald S, Morgan R, Hawkins K, Ssali S, George A, Molyneux S. The importance of gender analysis in research for health systems strengthening. Vol. 32, Health policy and planning. 2017. p. v1–3.

For peer review only - http://bmjopen.bmj.com/site/about/guidelines.xhtml

23. Tannenbaum C, Ellis RP, Eyssel F, Zou J, Schiebinger L. Sex and gender analysis improves science and engineering. Nature. 2019 Nov;575(7781):137–46.

24. More and Better Gender Data: A Powerful Tool for Improving Lives, The World Bank. 2016.

25. International Organization for Standardization 26000, Guidance on social responsibility.

26. DeCorby-Watson K, Mensah G, Bergeron K, Abdi S, Rempel B, Manson H. Effectiveness of capacity building interventions relevant to public health practice: a systematic review. BMC Public Health. 2018 Jun;18(1):684.

27. Bob Hayes. Machine Learning Adoption Rates Around the World. Business Broadway. 2021.

28. Artificial Intelligence and Start-Ups in Low- and Middle-Income Countries: Progress, Promises and Perils. Global System for Mobile Communications. 2020.

29. Bates DW, Saria S, Ohno-Machado L, Shah A, Escobar G. Big data in health care: using analytics to identify and manage high-risk and high-cost patients. Health Aff (Millwood). 2014 Jul;33(7):1123–31.

30. Shaoibi A, Neelon B, Lenert LA. Shared Decision Making: From Decision Science to Data Science. Med Decis Mak an Int J Soc Med Decis Mak. 2020 Apr;40(3):254–65.

For peer review only

For peer review only - http://bmjopen.bmj.com/site/about/guidelines.xhtml

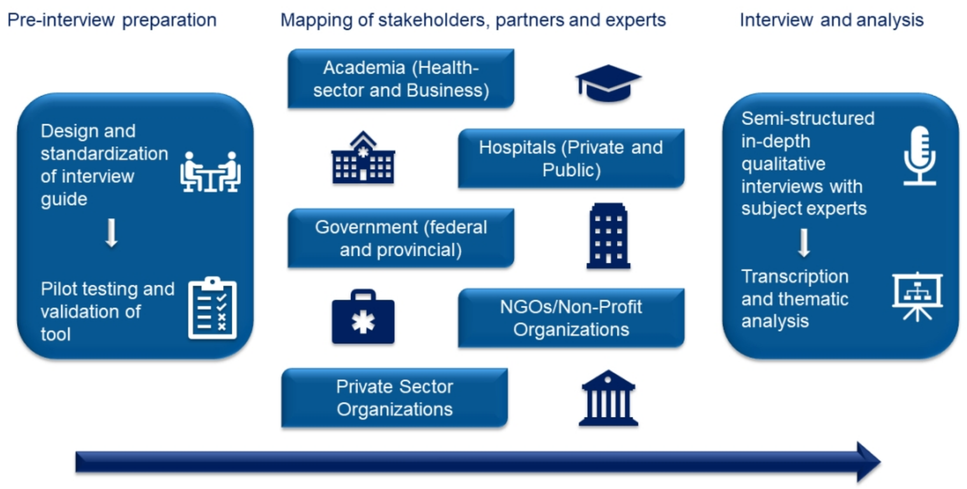

An overview of the methodological framework of the study – participant cohorts, process of interview preparation, conductance, and analysis

108x60mm (300 x 300 DPI)

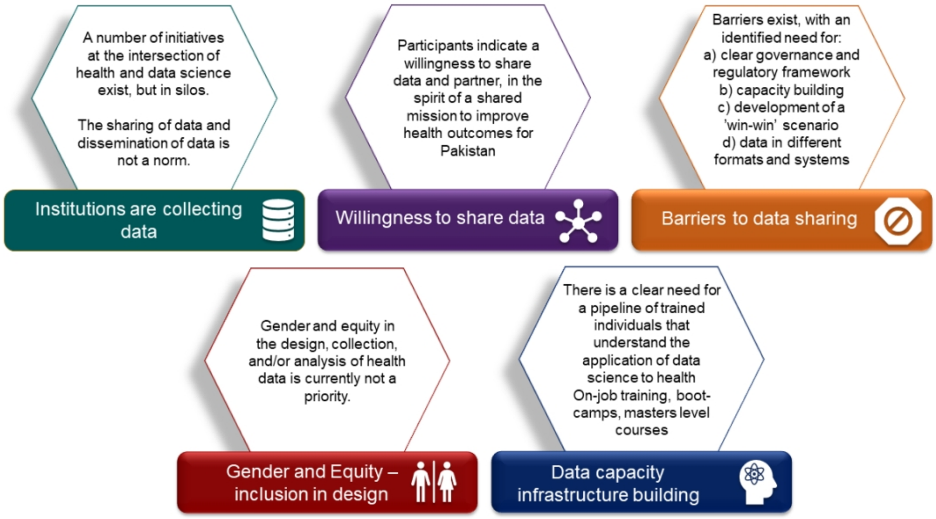

Key themes emerging from the thematic analysis and noteworthy takeaways

108x60mm (300 x 300 DPI)

Semi-structured interview guide

<u>Section 1 : Understanding the health data landscape for Pakistan</u>

What type of health data exists in Pakistan?

Potential prompts in case of a brief reply

- What type of data at a national/regional/global level supports your decision-making ability/research work?
- What type of health data would further support your ability to make informed decisions?
- Is health data at a Pakistan level accessible?
- Is health data at a Pakistan level of good quality? (define quality)

<u>Section 2: Understanding the application of a gender and equity lens to data</u>

Potential prompts in case of a brief reply (mostly guided by the interviewee's response)

- Do we know how to apply a gender/equity lens to our data (disaggregation, analysis etc)
- What population group do you not frequently see available data about?

<u>Section 3: Understanding the organizational handle on health data and its current role</u>

Potential prompts in case of a brief reply (mostly guided by the interviewee's response)

- What health data does your organization hold and to what level does your organization engage with the data for decision making.
- How equipped are organizations to manage the health data they hold?
- What kind of infrastructure/software does your organization have? Is it sufficient?

<u>Section 4: Understanding perceptions around developing a health data science training program/curriculum</u>

How effective do you think the introduction of a health data science training curriculum will be, to address barriers?

Potential prompts in case of a brief reply (mostly guided by the interviewee's response)

- What type of training in data science would be most beneficial to you and why?
- What health data science curriculum/training programs exist and are useful?
- Do you think there's an existing need for development of such a program in Pakistan? Why or why not?
- What barriers should such a training program address?
- What components should the health data science training curriculum have?

For peer review only

Consolidated criteria for reporting qualitative studies (COREQ): 32-item checklist

No. Item	Guide questions/description	Reported on Page #
**Domain 1: Research team and reflexivity**		
Personal Characteristics		
1. Inter viewer/facilitator	Which author/s conducted the interview or focus group?	8
2. Credentials	What were the researcher's credentials? E.g., PhD, MD	8
3. Occupation	What was their occupation at the time of the study?	8
4. Gender	Was the researcher male or female?	8
5. Experience and training	What experience or training did the researcher have?	8
Relationship with participants		
6. Relationship established	There was no personal relationship between interviewers	N/A
7. Participant knowledge of the interviewer	What did the participants know about the researcher? e.g., personal goals, reasons for doing the research	N/A
8. Interviewer characteristics	What characteristics were reported about the inter viewer/facilitator? e.g., Bias, assumptions, reasons and interests in the research topic	N/A

**Domain 2: study design**		
Theoretical framework		

For peer review only - http://bmjopen.bmj.com/site/about/guidelines.xhtml

9. Methodological orientation and Theory	What methodological orientation was stated to underpin the study? e.g., grounded theory, discourse analysis, ethnography, phenomenology, content analysis	8
Participant selection		
10. Sampling	How were participants selected? e.g., purposive, convenience, consecutive, snowball	8
11. Method of approach	How were participants approached? e.g., face-to-face, telephone, mail, email	8
12. Sample size	How many participants were in the study?	10
13. Non-participation	How many people refused to participate or dropped out? Reasons?	N/A
Setting		
14. Setting of data collection	Where was the data collected? e.g., home, clinic, workplace	8
15. Presence of non-participants	Was anyone else present besides the participants and researchers?	8
16. Description of sample	What are the important characteristics of the sample? e.g., demographic data, date	10
Data collection		
17. Interview guide	Were questions, prompts, guides provided by the authors? Was it pilot tested?	7
18. Repeat interviews	Were repeat inter views carried out? If yes, how many?	8
19. Audio/visual recording	Did the research use audio or visual recording to collect the data?	8

For peer review only - http://bmjopen.bmj.com/site/about/guidelines.xhtml

For peer review only

20. Field notes	Were field notes made during and/or after the interview or focus group?	N/A
21. Duration	What was the duration of the inter views or focus group?	8
22. Data saturation	Was data saturation discussed?	8
23. Transcripts returned	Were transcripts returned to participants for comment and/or correction?	N/A
**Domain 3: analysis and findings**		
Data analysis		
24. Number of data coders	How many data coders coded the data?	9
25. Description of the coding tree	Did authors provide a description of the coding tree?	N/A
26. Derivation of themes	Were themes identified in advance or derived from the data?	9
27. Software	What software, if applicable, was used to manage the data?	9
28. Participant checking	Did participants provide feedback on the findings?	N/A
Reporting		
29. Quotations presented	Were participant quotations presented to illustrate the themes/findings? Was each quotation identified? e.g., participant number	11-15
30. Data and findings consistent	Was there consistency between the data presented and the findings?	11-15
31. Clarity of major themes	Were major themes clearly presented in the findings?	11-15
32. Clarity of minor themes	Is there a description of diverse cases or discussion of minor themes?	N/A

For peer review only - http://bmjopen.bmj.com/site/about/guidelines.xhtml

For peer review only

For peer review only - http://bmjopen.bmj.com/site/about/guidelines.xhtml

Health Data Ecosystem in Pakistan – A Multi-sectoral Qualitative Assessment of Needs and Opportunities

Journal:	*BMJ Open*
Manuscript ID	bmjopen-2023-071616.R1
Article Type:	Original research
Date Submitted by the Author:	11-Jul-2023
Complete List of Authors:	Mahmood, Sana; Aga Khan University; Aga Khan University, Department of Community Health Sciences<br>Noorali, Ali Aahil; The Aga Khan University<br>Manji, Afshan; Aga Khan University, Department of Medicine; Aga Khan University<br>Afzal, Noreen; The Aga Khan University<br>Abbas, Saadia; Aga Khan University, Department of Medicine<br>Qamar, Javeria Bilal; Aga Khan University, Department of Medicine<br>Siddiqi, Sameen; Aga Khan University Medical College Pakistan, Community Health Sciences Department<br>Hoodbhoy, Zahra; Aga Khan University, Department of Pediatrics and Child Health<br>Virani, Salim; Michael E. DeBakey Veterans Affairs Medical Center HSR&D, Health Policy, Quality & Informatics Program,<br>Bhutta, Zulfiqar; Hosp Sick Children, Centre for Global Child Health<br>Samad, Zainab; The Aga Khan University; Duke Clinical Research Institute, Department of Medicine
**Primary Subject Heading**:	Public health
Secondary Subject Heading:	Qualitative research
Keywords:	QUALITATIVE RESEARCH, PUBLIC HEALTH, Quality in health care < HEALTH SERVICES ADMINISTRATION & MANAGEMENT

SCHOLARONE™
Manuscripts

I, the Submitting Author has the right to grant and does grant on behalf of all authors of the Work (as defined in the below author licence), an exclusive licence and/or a non-exclusive licence for contributions from authors who are: i) UK Crown employees; ii) where BMJ has agreed a CC-BY licence shall apply, and/or iii) in accordance with the terms applicable for US Federal Government officers or employees acting as part of their official duties; on a worldwide, perpetual, irrevocable, royalty-free basis to BMJ Publishing Group Ltd ("BMJ") its licensees and where the relevant Journal is co-owned by BMJ to the co-owners of the Journal, to publish the Work in this journal and any other BMJ products and to exploit all rights, as set out in our licence.

The Submitting Author accepts and understands that any supply made under these terms is made by BMJ to the Submitting Author unless you are acting as an employee on behalf of your employer or a postgraduate student of an affiliated institution which is paying any applicable article publishing charge ("APC") for Open Access articles. Where the Submitting Author wishes to make the Work available on an Open Access basis (and intends to pay the relevant APC), the terms of reuse of such Open Access shall be governed by a Creative Commons licence – details of these licences and which Creative Commons licence will apply to this Work are set out in our licence referred to above.

Other than as permitted in any relevant BMJ Author's Self Archiving Policies, I confirm this Work has not been accepted for publication elsewhere, is not being considered for publication elsewhere and does not duplicate material already published. I confirm all authors consent to publication of this Work and authorise the granting of this licence.

For peer review only

Health Data Ecosystem in Pakistan – A Multi-sectoral Qualitative Assessment of Needs and Opportunities

Authors: Sana Mahmood EdM [1,2,3], Ali Aahil Noorali MBBS [4,5], Afshan Manji MSc [4,5], Noreen Afzal MPhil [1], Saadia Abbas [4], Javeria Bilal Qamar [4], Sameen Siddiqi DrMed [2], Zahra Hoodbhoy PhD[6], Salim S. Virani PhD[7], Zulfiqar A. Bhutta, PhD [3], Zainab Samad MHS [3,4,8]

Affiliations:

[1] Dean's Office, Medical College, Aga Khan University, Karachi, Pakistan

[2] Department of Community Health Sciences, Aga Khan University, Karachi, Pakistan

[3] Institute of Global Health and Development, Aga Khan University, Karachi, Pakistan

[4] Department of Medicine, Medical College, Aga Khan University, Karachi, Pakistan

[5] CITRIC Health Data Science Center, Medical College, Aga Khan University, Karachi, Pakistan

[6] Department of Pediatrics and Child Health, Aga Khan University, Karachi, Pakistan

[7] Division of Cardiology, Department of Medicine, Baylor College of Medicine, USA

[8] Division of Cardiology, Department of Medicine, Duke University, Duke Global Health Institute, Duke Clinical Research Institute, Durham, NC

Corresponding Author:

Zainab Samad, MBBS, MHS

Aga Khan University, Stadium Road, Karachi, Pakistan, 74800

Email: samad.zainab@aku.edu

Phone: 03002017196

For peer review only

For peer review only - http://bmjopen.bmj.com/site/about/guidelines.xhtml

Word count:

4,255 words

For peer review only

For peer review only - http://bmjopen.bmj.com/site/about/guidelines.xhtml

Abstract

Objective:

Data are essential for tracking and monitoring of progress on health-related sustainable development goals (SDGs). But the capacity to analyze subnational and granular data is limited in low- and middle-income countries. Although Pakistan lags behind on achieving several health-related SDGs, its health information capacity is nascent. Through an exploratory qualitative approach, we aimed to understand the current landscape and perceptions on data in decision making among stakeholders of the health data ecosystem in Pakistan.

Design:

We used an exploratory qualitative study design.

Setting:

This study was conducted at the Aga Khan University, Karachi, Pakistan.

Participants:

We conducted semi-structured, in-depth interviews with multidisciplinary and multisectoral stakeholders from academia, hospital management, government, NGOs, and other relevant private entities till thematic saturation was achieved. Interviews were recorded and transcribed, followed by thematic analysis using NVivo.

Results:

Thematic analysis of 15 in-depth interviews revealed three major themes: 1) Institutions are collecting data, but face barriers to its effective utilization for decision making. These include lack of collection of needs-responsive data, lack of a gender/equity in data collection efforts, inadequate digitization, data reliability, and limited analytical ability; 2) There is openness and enthusiasm for sharing data for advancing health, however, multiple barriers hinder this

For peer review only - http://bmjopen.bmj.com/site/about/guidelines.xhtml

including appropriate regulatory frameworks, platforms for sharing data, inter-operability, and defined win-win scenarios; 3) There is limited capacity in the area of both human capital and infrastructure, for being able to use data to advance health but there is appetite to improve and invest in capacity in this area

Conclusions:

Our study identified key areas of focus that can contribute to orient a national health data roadmap and ecosystem in Pakistan.

For peer review only

For peer review only - http://bmjopen.bmj.com/site/about/guidelines.xhtml

Strengths and limitations of this study

- Our study is the first multi-disciplinary endeavor to explore perceptions around health data and health data science in Pakistan.
- We present perspectives from a low resource setting which has contextual relevancy and implications for other LMICs in the region.
- Our study participants were experts and decision makers from multiple sectors, across provinces, and with work at the intersection of health and data science.

  Our findings may not provide room for generalizability, beyond the Global South.

For peer review only

Introduction

Data are essential for tracking and monitoring progress on health-related sustainable development goals (SDGs).[1–3] While data and data analytics are being used in high income countries (HICs) to improve health equity, health outcomes, and continuously inform healthcare systems, their use in low-middle-income countries (LMICs) is lagging.[4–8] Investing in data ecosystems represents an important opportunity for monitoring and quickening progress on health-related SDGs in LMICs.[1,8,11,12]

With a population of 230 million, Pakistan, the fifth most populous LMIC, has a high estimated mortality and morbidity burden for various diseases, but its health system and health information system capacity is nascent.[13,14] However, during the COVID-19 pandemic, data were made nationally available in almost real-time, and data science methods were used to inform health policy and population level interventions such as smart lock downs and vaccinations efforts.[15,16] multi-stakeholder and interprovincial collaboration underpinned this successful effort and highlighted the need for a national health data ecosystem outside of crisis situations. To inform such future efforts, an understanding of the current perceptions around health data, its use in decision making and the health data ecosystem in Pakistan is required. To this end, we adopted a qualitative approach to understand the current landscape as well as perceptions on data in decision-making among a wide range of stakeholders.

For peer review only

For peer review only - http://bmjopen.bmj.com/site/about/guidelines.xhtml

Methods

Study Design and Setting:

This was an exploratory qualitative study with the primary objectives to comprehend the scope of the health data ecosystem in Pakistan, the knowledge and attitudes around developing partnerships and sharing data, and perceptions around the need for developing health data science capacity in Pakistan.

The study was led by investigators at the Aga Khan University (AKU) in Pakistan. With a forty-year presence in Pakistan, AKU has well established partnerships at both provincial and national levels, with government and academia, enabling regular engagement in interdisciplinary policy discussions and fora.

Study instrument:

A semi-structured interview guide was designed using carefully curated questions (available in supplement 1). The guide prompted a detailed discussion on the landscape and scope of existing health data. Further discussion was rooted in potential facilitators and barriers to building a national health data collaborative that would contribute to improved health outcomes in Pakistan. This included understanding the nature of existing policies and collaboratives, the availability and need of human capital for health data initiatives, and structures—from governance to infrastructure, which were present or would need to be developed and implemented to allow for organizations across sectors to comfortably share data to advance health outcomes in Pakistan.

The guide was pilot tested among a diverse cohort of four individuals and judged for clarity of questions. Feedback from the pilot testing was incorporated to address gaps in the interview

For peer review only - http://bmjopen.bmj.com/site/about/guidelines.xhtml

guide. Interviews were conducted by two female investigators (ZS and SM), who were the departmental chair and director, respectively, while the research staff (AAN, AA, SA, JBQ) acted as observers. Standardization was maintained across all interviews by ensuring that the same two interviewers conducted all the interviews with the same guide. Both interviewers had prior experience of conducting qualitative interviews. Each interview was conducted online for a duration that varied between 30 minutes and 2 hours.

Sampling, Inclusion and Exclusion Criteria:

A scoping exercise was conducted to identify experts and relevant institutions. Through discussion, the investigators collectively identified key sectors in the health ecosystem of Pakistan for a landscape analysis which formed the inclusion criteria: 1. University and academia with faculty in health and/or information technology (IT), 2. Senior-level hospital management (both private and public) 3. Government ministers (federal and provincial/state), 4. Non-governmental organizations (NGOs) and 5. Private-sector organizations (pharmaceutical, finance, and health insurance sectors). (Figure 1) There were no major or minor exclusion criteria.

Following convenience sampling to select key stakeholders with a particular focus on those with management/decision-making roles, invites were extended via email, and interviews were arranged. Thematic saturation was reached at 15 which comprised the final study sample. Based on the SDGs 4, 5 and 9 [17] (quality education, gender equality and industry, innovation and infrastructure respectively), we performed a mapping of major institutions across these domains

For peer review only - http://bmjopen.bmj.com/site/about/guidelines.xhtml

in the public and private health sectors, private organizations and NGOs. Major institutions were defined as those that were expected to have organizational maturity and scale in the area of capacity to collect data. Lastly, individuals with at least five years of leadership experience in their respective domains were eligible to participate in the study.

Data Analysis:

Grounded theory and the six-step method of thematic analysis of Braun and Clarke (2006) guided the analytical process.[18] Interviews were first audio recorded and transcribed verbatim. Since the research staff were not only observers, but also transcribers for the interviews, these roles helped them get familiarized and immersed with the collected data. Transcripts were imported into NVivo (version 12). An initial list of a priori codes was used by two research team members (AAN and AM) to code the transcripts. New codes emerging from the data, which were deemed relevant to the study objectives were also added to this list. This codebook was refined through iteration and consensus among research staff which helped in standardization of codes applied in all transcripts.

Data coded under similar codes were then grouped to identify major themes, which were paired with direct verbatim quotations from the interviewees. The themes were then reviewed to ensure adequate data and participant quotations supported the creation of each theme. All themes were then defined to convey an adequate description of its subthemes and relevant data. Lastly, the results were written in a format to describe the analyzed data.

Patient and Public Involvement

For peer review only - http://bmjopen.bmj.com/site/about/guidelines.xhtml

Patients and the public were not involved in the research design, analysis and dissemination of the findings.

Ethical Considerations:

The study received approval from the Ethical Review Committee at AKU (ERC # 2021-5839-16883). Written informed consent over email for the study was obtained from each participant before starting the interview.

Results

We conducted 15 in-depth interviews with a diverse range of stakeholders from five centralized cohorts. Sector and designation of the participants are described in Table 1. Thematic analysis generated three overarching themes: 1) Institutions are collecting data, but face barriers to its effective utilization for decision making. These include lack of collection of needs-responsive data, lack of a gender/equity in data collection efforts, inadequate digitization, data reliability, and limited analytical ability.; 2) There is openness and enthusiasm for sharing data for advancing health, however, multiple barriers hinder this endeavor; 3) There is limited capacity in the area of both human capital and infrastructure, for being able to use data to advance health but there is appetite to improve capacity in this area.

Table 1: Sector and designation of study participants (n=15)

**Sector**	**N (%)**
Academia	1
Hospitals	2
Government	3
Non-governmental organizations (NGOs)	3
Private-sector organizations	6
**Designation**	

For peer review only - http://bmjopen.bmj.com/site/about/guidelines.xhtml

For peer review only

Mid-level management	5
Chief medical officer	2
Health minister	2
Senior management	6

Theme 1: Institutions are collecting data, but face barriers to its effective utilization for decision making. These include lack of collection of needs-responsive data, lack of gender/equity in data collection efforts, inadequate digitization, data reliability and limited analytical ability.

Experts communicated that there are several initiatives at the intersection of health and data, and organizations are collecting and holding data, but these initiatives exist in silos. Institutions have a large volume of operational data, but several barriers to their effective utilization were identified. These ranged from lack of collection of needs-responsive data, inadequate digitization, inappropriate data formats, data reliability, value placed on ultimate data use by data collectors, and human capacity to gauge scientific insights from it.

Adequate data mapping and dataset awareness within institutions affecting access and use was identified as a barrier. An expert in digital health strategy noted that *"I would think that the dissemination of the data is a far bigger issue than the data holes being there. So, if you start digging, you find data sets, but you see that no one is aware of them, even though a lot of activity has happened"*.

Leaders identified lack of needs-responsive data collection as a barrier to effective utilization of data for decision making. A provincial health minister stated that, "*We find that getting population level information is really difficult. And you can get this information, but it's not really formalized - you just hear verbal estimates. So, in terms of planning, there is no common*

For peer review only - http://bmjopen.bmj.com/site/about/guidelines.xhtml

information base that people have that is like the gold standard." In a similar vein, a leader in a prominent healthcare institution noted, *"There are very few hospitals in Pakistan that actually record quality and patient safety data. So, hospital quality level data, the kind that exists in the NHS, or in the US etc. doesn't exist here."* In addition, most participants noted that gender and equity lens have not been widely considered, neither during the collection and analysis of health data, nor in the design of research and data driven initiatives. With regards to gender, a leader at a non-profit organization explained that: *"Disaggregated data by gender is probably a large problem nationally, particularly with development indicators."* Equity is often overlooked in conversations around data, and when considered, the level of commitment is inadequate. A governmental leader mentioned that: *"Unfortunately, I think equity is more a function of conversations with development partners. And it does translate to some commitment, but not the level of commitment that should be the case."*

The digitization and structure of available data is another holdup, as stated by a manager at a non-governmental organization: "*Some organizations don't have data in a format that's easily accessible, because they don't have electronic systems."* Most participants shared the perception that there is a lack of an appropriately designed system to help collate data to its most desired format. This sentiment was shared by another expert in epidemiology at a tertiary care hospital, who noted that, "*The reality was when I came in, I realized that the structure of the data being collected, was not conducive to be pulled out, right, so we couldn't do any research*." Another expert at an NGO noted, *"When I joined X organization, all this information was collected on different platforms, some of this was paper based, some of it was collected with in-house applications. And none of it was necessarily standards-based. And none of these applications were well designed for information sharing.*

For peer review only - http://bmjopen.bmj.com/site/about/guidelines.xhtml

Data reliability was another aspect highlighted in the interviews. Specifically in the context of data sharing, a leader at an NGO noted, "*You get conflicting information….who is going to verify whether database A is correct or database B is correct*"

A provincial minister also suggested that while data-driven initiatives do exist, there is sometimes lack of clarity regarding the purpose and objectives of successfully collecting data and ultimately value attached to these efforts among those collecting and managing this information. "*We tried to give the lady health workers an app to update in real time to update the data about the diseases that they are seeing and the pregnant women that they are seeing. We don't have a proper accountability system and so we cannot tell people that this is something important for health policy and interventions and for a database for knowledge.*"

Many interviewees felt that where data did exist, there were other obstacles like significant deficits in staffing and the inability to utilize the collected data. A leader in an NGO noted, "*We do not have trained people in the system, even people who have dealt with data for a long time, do not have the analytical skills to make sense of it, draw conclusions, and ask questions. That has been a real challenge.*"

Theme 2: There is openness and enthusiasm for sharing data for advancing health, however, multiple barriers hinder this including appropriate regulatory frameworks, platforms for sharing data, inter-operability, and defined win-win scenarios

Considering a shared vision to improve health outcomes in Pakistan, leaders indicated overall willingness to share data and partner for this common mission, expressing keen interest and

For peer review only - http://bmjopen.bmj.com/site/about/guidelines.xhtml

stating their openness to proposals and collaborations. Defining win-win scenarios would be critical to organizations sharing data. A government leader observed: *"I think we are open to proposals where, say, we make data in specific areas available. As long as we get something in return - if I can get more immediate impact out of that process, then that would excite me more. So you name an area where we can problem-solve, and we can actually close the loop on that partnership (in terms of how we can actually translate it to some impact)."* Interestingly, an epidemiologist at a tertiary care hospital shared insights about ensuring that a shared collaborative keeps 'democracy of data' as a central guiding principle: *"I think there has to be democracy of data sharing within an organization because there's no point hanging on to data, and not sharing it so that somebody can make use of it, and that is one of the problems; people hang on to data as a good treasure that they cannot share with anybody."*

Most experts shared that certain challenges and pertinent questions would need to be accounted for to build a sustainable, shared, accessible data ecosystem. These include the validity and accuracy of datasets themselves, privacy and regulatory framework around data sharing, addressing systemic differences between different sectors and inadequate workforce and training. Experts shared that even if the data exists, dissemination of data is always an issue because data governance is a nascent field in Pakistan. The term governance is also used broadly by interviewees that use it to refer to privacy and security of data as well as an overarching governing structure to establish appropriate ownership of data. An entity leader, at a premier bank, noted that this also holds true for other sectors of Pakistan, such as the well-resourced finance sector, where one of the country's largest banks is still working through the early stages of data governance. Furthermore, structuring a data system to be useful requires understanding what data fields one has in their database, what should go into those data fields, and a system that

For peer review only - http://bmjopen.bmj.com/site/about/guidelines.xhtml

is designed to ensure a clear utilitarian purpose. Though governance appears to be a major barrier for a data collaborative, subjects report that missing-ness and access to data are also impediments to utilization.

A manager and planning executive at a bank, noted: *"Some concerns in data sharing include who will own the data, what will be done with the data? Will the data remain valid or not, will transparency be maintained, privacy rules will be followed or not, ownership of the data? Where will data be used ultimately."*

Similarly, a chief medical officer at a tertiary hospital mentioned that: *"There needs to be a lot more structure put into data sharing, and by structure, what I mean is that rules and regulations (which need to be set up a priori). That will really give confidence to individual institutions and individuals who own that data - that their data is going to be used properly, reliably and honestly".*

Interoperability of data sets was reported as a big challenge due to differences in dataset formats and different data capacity/skills across different institutions and across public and private institutions. Issues of interoperability are further pronounced when complemented with differences in the approach of private vs public sectors and inpatient vs outpatient data. A leader in healthcare administration stated that *"Record keeping is something that is very poor there. In-patient record keeping is there, but there is nothing for out-patients at all. The private sector does keep the record, but the public sector does not. But the private sector does not share that data at all"*

For peer review only - http://bmjopen.bmj.com/site/about/guidelines.xhtml

Theme 3: There is limited capacity in the area of both human capital and infrastructure, for being able to use data to advance health but there is appetite to improve and invest in capacity in this area

Inadequate human resources in data management and analytical skills in organizations was identified as a major barrier to both effective internal use of data and external collaborative data sharing efforts. Participants remarked that data sharing through a collaborative, would require capacity-building in this area.

A manager at an NGO noted: *"The issue is not whether people are willing to share data. But certain organizations, traditional non-profit ones for example, don't have data teams or data managers (because of cost budgetary constraints). So, I think that these organizations often don't have the capacity to manage data."* It was also mentioned that lack of capacity building was a barrier to successfully analyzing and synthesizing data. A provincial healthcare leader noted: *"The main problem here is that we don't have an HR [human resource] there or a proper computerized system there to log in that data and upload it. We have a lot of restrictions in the IT department."* Similarly, a senior leader at a tertiary care hospital mentioned: "*We have the data, but we don't have the capacity and capability to analyze it and make changes in healthcare."* There is clear appetite and keen interest in investing organizational data capacity ranging from investing in needs-responsive data repositories and electronic systems, to upskilling the current workforce. A representative from academia shared their experience of setting up a data repository and its potential future impact: *"The long-term objective of this Higher Education Data Repository initiative is that we collect all the granular information throughout a student's*

For peer review only - http://bmjopen.bmj.com/site/about/guidelines.xhtml

life cycle. This will generate a lot of data for analysis, about what kind of educational needs we have in our system, what kind of courses do we need to specialize in for our students and the areas that our faculty members need to specialize in."

A representative from a large public sector tertiary care hospital reported their initiative of data automation and the barriers associated with it: *"We have begun an initiative at our hospital where we are starting to do automation of the data and that is happening ... That is also not working because our staffing there has been rejected so we are now trying to have a medical record system as an operative thing to see how the medical reforms will work."*

An NGO leader described the process, components, and importance of building a data-driven team – a cohesive unit of trained individuals that understand the application of data sciences to health. He stated: "*There are four skills that I think are important in building out a data team that we found. One is data engineering, which is just someone who can query databases, particularly complex databases...Then, I think the next skill that we found useful is analytics, which is how to develop dashboards. And that is a very easily trainable skill. So, the third skill that's (commonly) not there is knowing what dashboard to make. And the fourth skill is data science, which is basically being able to model data and that's an even rarer skill especially locally."*

A key skill within building capacity to manage and analyze data is the ability to effectively communicate results. A government health leader also delineated the importance of communication in building health data capacity which may be an essential, yet neglected, skill. She stated that, "*There must be a trigger factor that allows the person to do a communications*

For peer review only - http://bmjopen.bmj.com/site/about/guidelines.xhtml

strategy and awareness policy to send that message across. I think communication is very important. You specifically need to know how to communicate."

A Government leader also stated the need for and importance of on-the-job training by stating that "*For me, the single silver bullet is creating data management routines that act as on the job training for managers."* He added, *"... you need a data boot camp for health leaders, like that is weeklong. And that is sort of morning to night. And that's trying to basically break their thinking and get them to use differently the information that is available, because there is still a lot more information that is available."*

Discussion

Our study is the first, multi-disciplinary endeavor to understand perceptions on health data and health data science in Pakistan. The main finding from our qualitative analysis is that the scope of data science in health for advancing health outcomes, is far-reaching in Pakistan and likely in other LMICs where organizations have collected a great deal of data but are in the early stages of understanding how best to leverage and utilize this data. Furthermore, there is potential for establishing a health data ecosystem comprised of a health data collaborative with an appropriate governance structure, intentionality toward data design elements focusing on gender, equity, and needs-responsiveness, that is supported by appropriate capacity building initiatives. Our study findings suggest that while we are at nascent stages of using data to progress a national health agenda, several independent and national efforts are being made to allow for digitization and automation in healthcare and there is keen interest in investment in building capacity in this area. [19]Even though the far-reaching scope of health data and data science methods in healthcare

For peer review only - http://bmjopen.bmj.com/site/about/guidelines.xhtml

and their potential benefits are recognized by developing countries, development of a national data collaborative that might serve as a foundational block of a larger health data ecosystem is a complex endeavor and presents some challenges.[12] A principal lever for this agenda is timely access to the right information, but this has been a scarce resource in LMICs.[19]

Lack of rigorous and structured systems, problems with accuracy, credibility and completeness, inadequacy of trained personnel with core competencies, and unavailability of analytic tools were core obstacles highlighted by experts. Furthermore, there are very limited efforts to propagate a multimodal, multisectoral, and multidisciplinary approach to data, leading to a conspicuous lack of a central repository of information in LMICs like Pakistan.[8] In line with recent literature, a key concern highlighted by participants in data sharing was safeguarding their privacy, confidentiality, and security, with all interviewees agreeing to the need of a governance and regulatory framework being set up a priori to ensure data transparency and maintaining the trust of all parties involved that their data will be used honestly and reliably.[8,20]

Our participants also stated that the analysis, design, and collection of health data does not currently support gender and equity lens as the core of any organization. Disaggregation of data by gender is a problem nationally and is difficult to find. Literature suggests that health system policy development does not always pay adequate attention to gender and even when policies do include gender, intentions can evaporate when it comes to actual implementation.[21,22] Study participants suggested that equity was often a function of conversation with development partners and that the level of commitment to inclusion of equity needed to be increased. Tannenbaum et al. and The World Bank note that gender data is a powerful tool for improving lives as lack of disaggregated gender information has resulted in an incomplete disease understanding.[23,24] Furthermore, gender equity is an integral component of social

For peer review only - http://bmjopen.bmj.com/site/about/guidelines.xhtml

responsibility and according to International Organization for Standardization (ISO) 26000 Sustainable Development Goal 5 (Guidance on Social Responsibility), whereby the standard denotes the importance of having gender-inclusive leadership and governance in ensuring elimination of gender bias and promotion of gender parity.[25]

Our study participants mentioned how organizations collect and store data, have information management systems, but that data is not being utilized in the most effective way, due to limited capacity and skillsets. [26] Hence, they emphasized that translation and evidence synthesis require significant capacity building. A systematic review reflects on the importance of ongoing training and multilevel strategies needed in development of such programs, and how capacity building can influence different levels of entire organizations and systems.[27] The types of interventions assessed included internet-based teaching and workshops. The results of a worldwide cross-sectional survey by Kaggle et. al, illustrates the extent to which companies in various countries have adapted to machine learning models, with Israel surpassing even the United States.[28] . This need also represents an opportunity to develop local, contextual health data science programs that equip individuals with appropriate data management and data analytics skills[29].

A national strategy on establishing a robust health data ecosystem and data collaborative for Pakistan will be an important next step. This necessitates that the gaps identified globally and in our qualitative interviews are bridged and data are put into action. In this regard, a national health digital framework has recently been developed by the Ministry of Health, which can be used for developing a high-level roadmap. As noted by healthcare experts, the roadmap is to help healthcare professionals use data science principles to inform decision making, uplifting research, and guiding clinical approaches to improve healthcare delivery.[30,31]

For peer review only - http://bmjopen.bmj.com/site/about/guidelines.xhtml

This study has a few limitations. Our findings mainly stemming from interviews among leaders in the healthcare system of Pakistan do not provide such larger room for generalizability beyond the Global South. However, we present perspectives from a low resource setting which has contextual relevancy and implications for other LMICs in the region. Qualitative interviews focused on perspectives from key management leads at major institutions. This was primarily because the scope and objectives of this exercise were to assimilate input from experts and leaders in management, policy, and healthcare. A next step, on establishing a health data collaborative, will be to ensure data and perspectives from patients and communities, who serve as key stakeholders in healthcare systems.

Conclusion

The present study highlights important opportunities and barriers that need to be addressed to develop a health data ecosystem in Pakistan. Creation of appropriate governance, regulatory frameworks, gender and equity indicators, and defining win-win scenarios, are important principles to consider for planning any national health data collaboratives. To enable this ecosystem, collaboration is required on strategic outlining of how data can be collated, organized, curated, updated, and finally pipelined. For achieving this goal, building data science capacity within organizations would be critical, thus providing the ability to leverage health data to its full potential for informed decision making.

For peer review only - http://bmjopen.bmj.com/site/about/guidelines.xhtml

Contributors

All authors confirm that they had full access to all the data in the study and accept responsibility to submit for publication.

ZS conceived of, designed the study, collected data, and acquired funding.

SM collected the data and wrote the original draft of the manuscript.

AAN analyzed and interpreted the data and wrote the original draft of the manuscript.

AM analyzed and interpreted the data and wrote the original draft of the manuscript.

NA wrote the original draft of the manuscript.

SA was involved in data curation and manuscript writing.

JQB was involved in data curation and manuscript writing.

SS wrote the original draft of the manuscript.

ZH wrote the original draft of the manuscript.

ZAB wrote the original draft of the manuscript.

SSV wrote the original draft of the manuscript.

All authors contributed to critically reviewing and editing the manuscript.

For peer review only

Declaration of interests

We declare no competing interests.

Funding

For peer review only - http://bmjopen.bmj.com/site/about/guidelines.xhtml

This work was supported through a grant from the Bill & Melinda Gates Foundation (grant number: INV-021944).

Data availability statement

No data are available.

Ethics approval

The study received approval from the Ethical Review Committee at AKU (ERC # 2021-5839-16883).

License statement

I, the Submitting Author has the right to grant and does grant on behalf of all authors of the Work (as defined in the below author license), an exclusive license and/or a non-exclusive license for contributions from authors who are: i) UK Crown employees; ii) where BMJ has agreed a CC-BY license shall apply, and/or iii) in accordance with the terms applicable for US Federal Government officers or employees acting as part of their official duties; on a worldwide, perpetual, irrevocable, royalty-free basis to BMJ Publishing Group Ltd ("BMJ") its licensees and where the relevant Journal is co-owned by BMJ to the co-owners of the Journal, to publish the Work in BMJ Open and any other BMJ products and to exploit all rights, as set out in our license.

The Submitting Author accepts and understands that any supply made under these terms is made by BMJ to the Submitting Author unless you are acting as an employee on behalf of your

For peer review only

For peer review only - http://bmjopen.bmj.com/site/about/guidelines.xhtml

employer or a postgraduate student of an affiliated institution which is paying any applicable article publishing charge ("APC") for Open Access articles. Where the Submitting Author wishes to make the Work available on an Open Access basis (and intends to pay the relevant APC), the terms of reuse of such Open Access shall be governed by a Creative Commons license – details of these licenses and which Creative Commons license will apply to this Work are set out in our license referred to above.

Figure legend

Figure 1: An overview of the methodological framework of the study – participant cohorts, process of interview preparation, conductance, and analysis

For peer review only

References

1 Measuring progress from 1990 to 2017 and projecting attainment to 2030 of the health-related Sustainable Development Goals for 195 countries and territories: a systematic analysis for the Global Burden of Disease Study 2017. *Lancet (London, England)* 2018;**392**:2091–138. doi:10.1016/S0140-6736(18)32281-5

2 Transforming our world: the 2030 Agenda for Sustainable Development | Department of Economic and Social Affairs. https://sdgs.un.org/2030agenda (accessed 21 Nov 2022).

3 Sachs JD, Schmidt-Traub G, Mazzucato M, *et al.* Six Transformations to achieve the Sustainable Development Goals. *Nat Sustain 2019 29* 2019;**2**:805–14. doi:10.1038/s41893-019-0352-9

4 Bhavnani SP, Muñoz D, Bagai A. Data Science in Healthcare: Implications for Early Career Investigators. *Circ Cardiovasc Qual Outcomes* 2016;**9**:683–7. doi:10.1161/CIRCOUTCOMES.116.003081

5 Sharma A, Harrington RA, McClellan MB, *et al.* Using Digital Health Technology to Better Generate Evidence and Deliver Evidence-Based Care. *J Am Coll Cardiol* 2018;**71**:2680–90. doi:10.1016/j.jacc.2018.03.523

6 Ting DSW, Carin L, Dzau V, *et al.* Digital technology and COVID-19. *Nat Med 2020 264* 2020;**26**:459–61. doi:10.1038/s41591-020-0824-5

7 Imoto S, Hasegawa T, Yamaguchi R. Data science and precision health care. *Nutr Rev* 2020;**78**:53–7. doi:10.1093/nutrit/nuaa110

For peer review only - http://bmjopen.bmj.com/site/about/guidelines.xhtml

8 Bezuidenhout L, Chakauya E. Hidden concerns of sharing research data by low/middle-income country scientists. *Glob Bioeth = Probl di Bioet* 2018;**29**:39–54. doi:10.1080/11287462.2018.1441780

9 Ting DSW, Carin L, Dzau V, *et al.* Digital technology and COVID-19. *Nat Med* 2020;**26**:459–61. doi:10.1038/s41591-020-0824-5

10 Andreu-Perez J, Poon CCY, Merrifield RD, *et al.* Big data for health. *IEEE J Biomed Heal informatics* 2015;**19**:1193–208. doi:10.1109/JBHI.2015.2450362

11 Wyber R, Vaillancourt S, Perry W, *et al.* Big data in global health: improving health in low- and middle-income countries. *Bull World Health Organ* 2015;**93**:203–8. doi:10.2471/BLT.14.139022

12 Naseem M, Akhund R, Arshad H, *et al.* Exploring the Potential of Artificial Intelligence and Machine Learning to Combat COVID-19 and Existing Opportunities for LMIC: A Scoping Review. *J Prim Care Community Health* 2020;**11**:2150132720963634. doi:10.1177/2150132720963634

13 Brief on Census -2017 | Pakistan Bureau of Statistics. https://www.pbs.gov.pk/content/brief-census-2017 (accessed 21 Nov 2022).

14 Roth GA, Abate D, Abate KH, *et al.* Global, regional, and national age-sex-specific mortality for 282 causes of death in 195 countries and territories, 1980–2017: a systematic analysis for the Global Burden of Disease Study 2017. *Lancet* 2018;**392**:1736–88.http://www.thelancet.com/article/S0140673618322037/fulltext (accessed 21 Nov 2022).

15 Akhtar H, Afridi M, Akhtar S, *et al.* Pakistan's Response to COVID-19: Overcoming National and International Hypes to Fight the Pandemic. *JMIR Public Heal Surveill*

For peer review only

For peer review only - http://bmjopen.bmj.com/site/about/guidelines.xhtml

2021;**7**. doi:10.2196/28517

16 Haq IU, Rehman ZU. Medical Research in Pakistan; A Bibliometric Evaluation from 2001 to 2020. *Libr Philos Pract (e-journal*https://www.pmc.gov.pk/ (accessed 9 Dec 2022).

17 THE 17 GOALS | Sustainable Development. https://sdgs.un.org/goals (accessed 5 Jul 2023).

18 Braun V, Clarke V. Thematic analysis. *APA Handb Res methods Psychol Vol 2 Res Des Quant Qual Neuropsychol Biol* 2012;:57–71. doi:10.1037/13620-004

19 Harrison K, Rahimi N, Danovaro-Holliday MC. Factors limiting data quality in the expanded programme on immunization in low and middle-income countries: A scoping review. *Vaccine* 2020;**38**:4652–63. doi:10.1016/j.vaccine.2020.02.091

20 Tiffin N, George A, LeFevre AE. How to use relevant data for maximal benefit with minimal risk: digital health data governance to protect vulnerable populations in low-income and middle-income countries. *BMJ Glob Heal* 2019;**4**:e001395. doi:10.1136/bmjgh-2019-001395

21 Morgan R, Ayiasi RM, Barman D, *et al.* Gendered health systems: evidence from low- and middle-income countries. *Heal Res policy Syst* 2018;**16**:58. doi:10.1186/s12961-018-0338-5

22 Theobald S, Morgan R, Hawkins K, *et al.* The importance of gender analysis in research for health systems strengthening. Health Policy Plan. 2017;**32**:v1–3. doi:10.1093/heapol/czx163

23 Tannenbaum C, Ellis RP, Eyssel F, *et al.* Sex and gender analysis improves science and engineering. *Nature* 2019;**575**:137–46. doi:10.1038/s41586-019-1657-6

24 More and Better Gender Data: A Powerful Tool for Improving Lives, The World Bank.

For peer review only - http://bmjopen.bmj.com/site/about/guidelines.xhtml

2016.

25 International Organization for Standardization 26000, Guidance on social responsibility.

26 Artificial Intelligence and Start-Ups in Low- and Middle-Income Countries: Progress, Promises and Perils. 2020.

27 DeCorby-Watson K, Mensah G, Bergeron K, *et al.* Effectiveness of capacity building interventions relevant to public health practice: a systematic review. *BMC Public Health* 2018;**18**:684. doi:10.1186/s12889-018-5591-6

28 Bob Hayes. Machine Learning Adoption Rates Around the World. Bus. Broadw. 2021.

29 Hoodbhoy Z, Chunara R, Waljee A, *et al.* Is there a need for graduate-level programmes in health data science? A perspective from Pakistan. *Lancet Glob Heal* 2023;**11**:e23–5. doi:10.1016/S2214-109X(22)00459-4

30 Bates DW, Saria S, Ohno-Machado L, *et al.* Big data in health care: using analytics to identify and manage high-risk and high-cost patients. *Health Aff (Millwood)* 2014;**33**:1123–31. doi:10.1377/hlthaff.2014.0041

31 Shaoibi A, Neelon B, Lenert LA. Shared Decision Making: From Decision Science to Data Science. *Med Decis Mak an Int J Soc Med Decis Mak* 2020;**40**:254–65. doi:10.1177/0272989X20903267

For peer review only

For peer review only - http://bmjopen.bmj.com/site/about/guidelines.xhtml

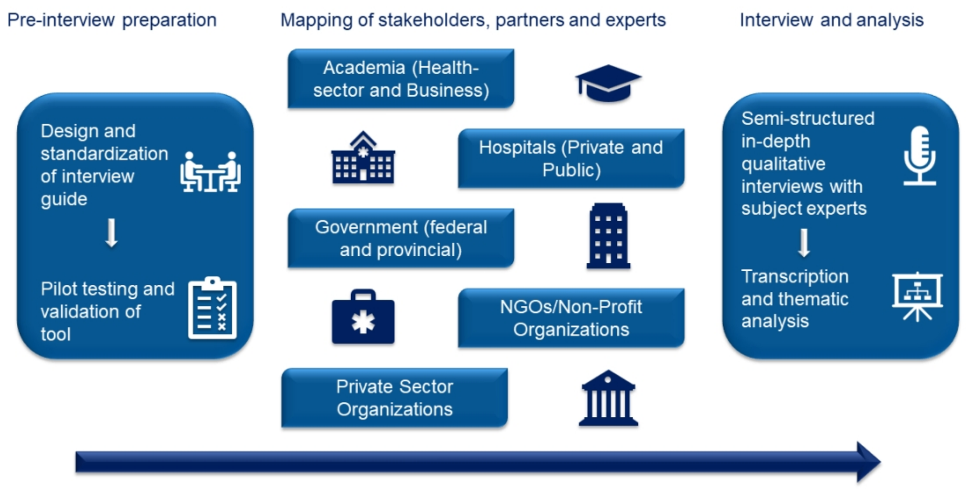

An overview of the methodological framework of the study – participant cohorts, process of interview preparation, conductance, and analysis

108x60mm (300 x 300 DPI)

Semi-structured interview guide

<u>Section 1 : Understanding the health data landscape for Pakistan</u>

What type of health data exists in Pakistan?

Potential prompts in case of a brief reply

- What type of data at a national/regional/global level supports your decision-making ability/research work?
- What type of health data would further support your ability to make informed decisions?
- Is health data at a Pakistan level accessible?
- Is health data at a Pakistan level of good quality? (define quality)

<u>Section 2: Understanding the application of a gender and equity lens to data</u>

Potential prompts in case of a brief reply (mostly guided by the interviewee's response)

- Do we know how to apply a gender/equity lens to our data (disaggregation, analysis etc)
- What population group do you not frequently see available data about?

<u>Section 3: Understanding the organizational handle on health data and its current role</u>

Potential prompts in case of a brief reply (mostly guided by the interviewee's response)

- What health data does your organization hold and to what level does your organization engage with the data for decision making.
- How equipped are organizations to manage the health data they hold?
- What kind of infrastructure/software does your organization have? Is it sufficient?

<u>Section 4: Understanding perceptions around developing a health data science training program/curriculum</u>

How effective do you think the introduction of a health data science training curriculum will be, to address barriers?

Potential prompts in case of a brief reply (mostly guided by the interviewee's response)

- What type of training in data science would be most beneficial to you and why?
- What health data science curriculum/training programs exist and are useful?
- Do you think there's an existing need for development of such a program in Pakistan? Why or why not?
- What barriers should such a training program address?
- What components should the health data science training curriculum have?

For peer review only

Consolidated criteria for reporting qualitative studies (COREQ): 32-item checklist

No. Item	Guide questions/description	Reported on Page #
**Domain 1: Research team and reflexivity**		
Personal Characteristics		
1. Inter viewer/facilitator	Which author/s conducted the interview or focus group?	7
2. Credentials	What were the researcher's credentials? E.g., PhD, MD	8
3. Occupation	What was their occupation at the time of the study?	8
4. Gender	Was the researcher male or female?	7
5. Experience and training	What experience or training did the researcher have?	8
Relationship with participants		
6. Relationship established	There was no personal relationship between interviewers	N/A
7. Participant knowledge of the interviewer	What did the participants know about the researcher? e.g., personal goals, reasons for doing the research	N/A
8. Interviewer characteristics	What characteristics were reported about the inter viewer/facilitator? e.g., Bias, assumptions, reasons and interests in the research topic	N/A

**Domain 2: study design**		
Theoretical framework		

For peer review only - http://bmjopen.bmj.com/site/about/guidelines.xhtml

9. Methodological orientation and Theory	What methodological orientation was stated to underpin the study? e.g., grounded theory, discourse analysis, ethnography, phenomenology, content analysis	9
Participant selection		
10. Sampling	How were participants selected? e.g., purposive, convenience, consecutive, snowball	8
11. Method of approach	How were participants approached? e.g., face-to-face, telephone, mail, email	8
12. Sample size	How many participants were in the study?	10
13. Non-participation	How many people refused to participate or dropped out? Reasons?	N/A
Setting		
14. Setting of data collection	Where was the data collected? e.g., home, clinic, workplace	8
15. Presence of non-participants	Was anyone else present besides the participants and researchers?	8
16. Description of sample	What are the important characteristics of the sample? e.g., demographic data, date	10
Data collection		
17. Interview guide	Were questions, prompts, guides provided by the authors? Was it pilot tested?	7
18. Repeat interviews	Were repeat inter views carried out? If yes, how many?	N/A
19. Audio/visual recording	Did the research use audio or visual recording to collect the data?	9

For peer review only

For peer review only - http://bmjopen.bmj.com/site/about/guidelines.xhtml

20. Field notes	Were field notes made during and/or after the interview or focus group?	N/A
21. Duration	What was the duration of the inter views or focus group?	8
22. Data saturation	Was data saturation discussed?	8
23. Transcripts returned	Were transcripts returned to participants for comment and/or correction?	N/A
**Domain 3: analysis and findings**		
Data analysis		
24. Number of data coders	How many data coders coded the data?	9
25. Description of the coding tree	Did authors provide a description of the coding tree?	N/A
26. Derivation of themes	Were themes identified in advance or derived from the data?	9
27. Software	What software, if applicable, was used to manage the data?	9
28. Participant checking	Did participants provide feedback on the findings?	N/A
Reporting		
29. Quotations presented	Were participant quotations presented to illustrate the themes/findings? Was each quotation identified? e.g., participant number	11-17
30. Data and findings consistent	Was there consistency between the data presented and the findings?	10-17
31. Clarity of major themes	Were major themes clearly presented in the findings?	10-17
32. Clarity of minor themes	Is there a description of diverse cases or discussion of minor themes?	N/A

For peer review only

For peer review only

For peer review only - http://bmjopen.bmj.com/site/about/guidelines.xhtml

BMJ Open

Health Data Ecosystem in Pakistan – A Multi-sectoral Qualitative Assessment of Needs and Opportunities

Journal:	*BMJ Open*
Manuscript ID	bmjopen-2023-071616.R2
Article Type:	Original research
Date Submitted by the Author:	20-Aug-2023

Complete List of Authors:	Mahmood, Sana; Aga Khan University; Aga Khan University, Department of Community Health Sciences<br>Noorali, Ali Aahil; The Aga Khan University<br>Manji, Afshan; Aga Khan University, Department of Medicine; Aga Khan University<br>Afzal, Noreen; The Aga Khan University<br>Abbas, Saadia; Aga Khan University, Department of Medicine<br>Qamar, Javeria Bilal; Aga Khan University, Department of Medicine<br>Siddiqi, Sameen; Aga Khan University Medical College Pakistan, Community Health Sciences Department<br>Hoodbhoy, Zahra; Aga Khan University, Department of Pediatrics and Child Health<br>Virani, Salim; Michael E. DeBakey Veterans Affairs Medical Center HSR&D, Health Policy, Quality & Informatics Program,<br>Bhutta, Zulfiqar; Hosp Sick Children, Centre for Global Child Health<br>Samad, Zainab; The Aga Khan University; Duke Clinical Research Institute, Department of Medicine

**Primary Subject Heading**:	Public health
Secondary Subject Heading:	Qualitative research
Keywords:	QUALITATIVE RESEARCH, PUBLIC HEALTH, Quality in health care < HEALTH SERVICES ADMINISTRATION & MANAGEMENT

SCHOLARONE™
Manuscripts

I, the Submitting Author has the right to grant and does grant on behalf of all authors of the Work (as defined in the below author licence), an exclusive licence and/or a non-exclusive licence for contributions from authors who are: i) UK Crown employees; ii) where BMJ has agreed a CC-BY licence shall apply, and/or iii) in accordance with the terms applicable for US Federal Government officers or employees acting as part of their official duties; on a worldwide, perpetual, irrevocable, royalty-free basis to BMJ Publishing Group Ltd ("BMJ") its licensees and where the relevant Journal is co-owned by BMJ to the co-owners of the Journal, to publish the Work in this journal and any other BMJ products and to exploit all rights, as set out in our licence.

The Submitting Author accepts and understands that any supply made under these terms is made by BMJ to the Submitting Author unless you are acting as an employee on behalf of your employer or a postgraduate student of an affiliated institution which is paying any applicable article publishing charge ("APC") for Open Access articles. Where the Submitting Author wishes to make the Work available on an Open Access basis (and intends to pay the relevant APC), the terms of reuse of such Open Access shall be governed by a Creative Commons licence – details of these licences and which Creative Commons licence will apply to this Work are set out in our licence referred to above.

Other than as permitted in any relevant BMJ Author's Self Archiving Policies, I confirm this Work has not been accepted for publication elsewhere, is not being considered for publication elsewhere and does not duplicate material already published. I confirm all authors consent to publication of this Work and authorise the granting of this licence.

For peer review only

Health Data Ecosystem in Pakistan – A Multi-sectoral Qualitative Assessment of Needs and Opportunities

Authors: Sana Mahmood EdM [1,2,3], Ali Aahil Noorali MBBS [4,5], Afshan Manji MSc [4,5], Noreen Afzal MPhil [1], Saadia Abbas [4], Javeria Bilal Qamar [4], Sameen Siddiqi DrMed [2], Zahra Hoodbhoy PhD[6], Salim S. Virani PhD[7], Zulfiqar A. Bhutta, PhD [3], Zainab Samad MHS [3,4,8]

Affiliations:

[1] Dean's Office, Medical College, Aga Khan University, Karachi, Pakistan

[2] Department of Community Health Sciences, Aga Khan University, Karachi, Pakistan

[3] Institute of Global Health and Development, Aga Khan University, Karachi, Pakistan

[4] Department of Medicine, Medical College, Aga Khan University, Karachi, Pakistan

[5] CITRIC Health Data Science Center, Medical College, Aga Khan University, Karachi, Pakistan

[6] Department of Pediatrics and Child Health, Aga Khan University, Karachi, Pakistan

[7] Division of Cardiology, Department of Medicine, Baylor College of Medicine, USA

[8] Division of Cardiology, Department of Medicine, Duke University, Duke Global Health Institute, Duke Clinical Research Institute, Durham, NC

Corresponding Author:

Zainab Samad, MBBS, MHS

Aga Khan University, Stadium Road, Karachi, Pakistan, 74800

Email: samad.zainab@aku.edu

Phone: 03002017196

For peer review only - http://bmjopen.bmj.com/site/about/guidelines.xhtml

For peer review only

Word count:

4,412 words

For peer review only

For peer review only - http://bmjopen.bmj.com/site/about/guidelines.xhtml

Abstract

Objective:

Data are essential for tracking and monitoring of progress on health-related sustainable development goals (SDGs). But the capacity to analyze subnational and granular data is limited in low- and middle-income countries. Although Pakistan lags behind on achieving several health-related SDGs, its health information capacity is nascent. Through an exploratory qualitative approach, we aimed to understand the current landscape and perceptions on data in decision making among stakeholders of the health data ecosystem in Pakistan.

Design:

We used an exploratory qualitative study design.

Setting:

This study was conducted at the Aga Khan University, Karachi, Pakistan.

Participants:

We conducted semi-structured, in-depth interviews with multidisciplinary and multisectoral stakeholders from academia, hospital management, government, NGOs, and other relevant private entities till thematic saturation was achieved. Interviews were recorded and transcribed, followed by thematic analysis using NVivo.

Results:

Thematic analysis of 15 in-depth interviews revealed three major themes: 1) Institutions are collecting data, but face barriers to its effective utilization for decision making. These include lack of collection of needs-responsive data, lack of a gender/equity in data collection efforts, inadequate digitization, data reliability, and limited analytical ability; 2) There is openness and enthusiasm for sharing data for advancing health, however, multiple barriers hinder this

For peer review only - http://bmjopen.bmj.com/site/about/guidelines.xhtml

including appropriate regulatory frameworks, platforms for sharing data, inter-operability, and defined win-win scenarios; 3) There is limited capacity in the area of both human capital and infrastructure, for being able to use data to advance health but there is appetite to improve and invest in capacity in this area

Conclusions:

Our study identified key areas of focus that can contribute to orient a national health data roadmap and ecosystem in Pakistan.

For peer review only

For peer review only - http://bmjopen.bmj.com/site/about/guidelines.xhtml

Strengths and limitations of this study

- Our study participants were experts and decision makers from multiple sectors, across provinces, and with work at the intersection of health and data science. In-depth interviews with key-informants allowed for a thorough exploration of the scope and challenges for health data science in Pakistan.
- We did not conduct patient interviews to learn about their opinions about the application of health data science in Pakistan.

For peer review only

For peer review only - http://bmjopen.bmj.com/site/about/guidelines.xhtml

Introduction

Data are essential for tracking and monitoring progress on health-related sustainable development goals (SDGs).[1–3] While data and data analytics are being used in high income countries (HICs) to improve health equity, health outcomes, and continuously inform healthcare systems, their use in low-middle-income countries (LMICs) is lagging.[4–8] Investing in data ecosystems represents an important opportunity for monitoring and quickening progress on health-related SDGs in LMICs.[1,8–10]

With a population of 230 million, Pakistan, the fifth most populous LMIC, has a high estimated mortality and morbidity burden for various diseases, but its health system and health information system capacity is nascent.[11,12] However, during the COVID-19 pandemic, data were made nationally available in almost real-time, and data science methods were used to inform health policy and population level interventions such as smart lock downs and vaccinations efforts.[13,14] Multi-stakeholder and interprovincial collaboration underpinned this successful effort and highlighted the need for a national health data ecosystem outside of crisis situations. To inform such future efforts, an understanding of the current perceptions around health data, its use in decision making and the health data ecosystem in Pakistan is required. To this end, we adopted a qualitative approach to understand the current landscape as well as perceptions on data in decision-making among a wide range of stakeholders.

For peer review only - http://bmjopen.bmj.com/site/about/guidelines.xhtml

Methods

Study Design and Setting:

This was an exploratory qualitative study with the primary objectives to comprehend the scope of the health data ecosystem in Pakistan, knowledge and attitudes around developing partnerships and sharing data, and perceptions around the need for developing health data science capacity in Pakistan.

The study was led by investigators at the Aga Khan University (AKU) in Pakistan. With a forty-year presence in Pakistan, AKU has well established partnerships at both provincial and national levels, with government and academia, enabling regular engagement in interdisciplinary policy discussions and fora.

Study instrument:

A semi-structured interview guide was designed using carefully curated questions (available in supplement 1). The guide prompted a detailed discussion on the landscape and scope of existing health data. Further discussion was rooted in potential facilitators and barriers to building a national health data collaborative that would contribute to improved health outcomes in Pakistan. This included understanding the nature of existing policies and collaboratives, the availability and need of human capital for health data initiatives, and structures—from governance to infrastructure, which were present or would need to be developed and implemented to allow for organizations across sectors to comfortably share data to advance health outcomes in Pakistan.

The guide was pilot-tested among a diverse cohort of four individuals and judged for clarity of questions. Feedback from the pilot testing was incorporated to address gaps in the interview

For peer review only - http://bmjopen.bmj.com/site/about/guidelines.xhtml

guide. Interviews were conducted by two female investigators (ZS and SM), who were the departmental chair and director, respectively, while the research staff (AAN, AA, SA, JBQ) acted as observers. Standardization was maintained across all interviews by ensuring that the same two interviewers conducted all the interviews with the same guide. Both interviewers had prior experience of conducting qualitative interviews. Each interview was conducted online for a duration that varied between 30 minutes and 2 hours.

Sampling, Inclusion and Exclusion Criteria:

A scoping exercise was conducted to identify experts and relevant institutions. Through discussion, the investigators collectively identified key sectors in the health ecosystem of Pakistan for a landscape analysis which formed the inclusion criteria: 1. University and academia with faculty in health and/or information technology (IT), 2. Senior-level hospital management (both private and public) 3. Government ministers (federal and provincial/state), 4. Non-governmental organizations (NGOs) and 5. Private-sector organizations (pharmaceutical, finance, and health insurance sectors). There were no major or minor exclusion criteria.

Based on the SDGs 4, 5 and 9 [15] (quality education, gender equality and industry, innovation and infrastructure respectively), we performed a mapping of major institutions across these domains in the public and private health sectors, private organizations and NGOs. Major institutions were defined as those that were expected to have organizational maturity and scale in the area and capacity to collect data. Lastly, individuals with at least five years of leadership experience in their respective domains were eligible to participate in the study.

For peer review only - http://bmjopen.bmj.com/site/about/guidelines.xhtml

For peer review only

Following convenience sampling to select key stakeholders with a particular focus on those with management/decision-making roles, invites were extended via email, and interviews were arranged. Thematic saturation was reached at 15 interviews which comprised the final study sample. Figure 1 illustrates the study methodology.

Data Analysis:

Grounded theory and the six-step method of thematic analysis of Braun and Clarke (2006) guided the analytical process.[16] Interviews were first audio recorded and transcribed verbatim. Since the research staff were not only observers, but also transcribers for the interviews, these roles helped them get familiarized and immersed with the collected data. Transcripts were imported into NVivo (version 12). An initial list of a priori codes was used by two research team members (AAN and AM) to code the transcripts. New codes emerging from the data, which were deemed relevant to the study objectives were also added to this list. This codebook was refined through iteration and consensus among research staff which helped in standardization of the codes applied in all transcripts.

Data coded under similar codes were then grouped to identify major themes, which were paired with direct verbatim quotations from the interviewees. The themes were then reviewed to ensure adequate data and participant quotations supported the creation of each theme. All themes were then defined to convey an adequate description of its subthemes and relevant data. Lastly, the results were written in a format to describe the analyzed data in detail.

Patient and Public Involvement

For peer review only - http://bmjopen.bmj.com/site/about/guidelines.xhtml

Patients and the public were not involved in the research design, analysis, and dissemination of the findings.

Ethical Considerations:

The study received approval from the Ethical Review Committee at AKU (ERC # 2021-5839-16883). Written informed consent over email for the study was obtained from each participant before starting the interview.

Results

We conducted 15 in-depth interviews with a diverse range of stakeholders from five centralized cohorts. Sector and designation of the participants are described in Table 1. Thematic analysis generated three overarching themes: 1) Institutions are collecting data, but face barriers to its effective utilization for decision making. These include lack of collection of needs-responsive data, lack of a gender/equity in data collection efforts, inadequate digitization, data reliability, and limited analytical ability.; 2) There is openness and enthusiasm for sharing data for advancing health, however, multiple barriers hinder this endeavor; 3) There is limited capacity in the area of both human capital and infrastructure, for being able to use data to advance health but there is appetite to improve capacity in this area.

**Table 1: Sector and designation of study participants (n=15)**	
**Sector**	**N (%)**
Academia	1
Hospitals	2
Government	3
Non-governmental organizations (NGOs)	3
Private-sector organizations	6
**Designation**	

For peer review only - http://bmjopen.bmj.com/site/about/guidelines.xhtml

Mid-level management	5
Chief medical officer	2
Health minister	2
Senior management	6

Theme 1: Institutions are collecting data, but face barriers to its effective utilization for decision making. These include lack of collection of needs-responsive data, lack of gender/equity in data collection efforts, inadequate digitization, data reliability and limited analytical ability.

Experts communicated that there are several initiatives at the intersection of health and data, and organizations are collecting and holding data, but these initiatives exist in silos. Institutions have a large volume of operational data, but several barriers to their effective utilization were identified. These ranged from lack of collection of needs-responsive data (data not capturing variables required for decision making, interventions and policy reform), inadequate digitization, inappropriate data formats, data reliability, value placed on ultimate data use by data collectors, and human capacity to gauge scientific insights from it.

Inadequate data mapping and lack of dataset awareness within institutions affecting access and use were identified as barriers. An expert in digital health strategy noted that *"I would think that the dissemination of the data is a far bigger issue than the data holes being there. So, if you start digging, you find data sets, but you see that no one is aware of them, even though a lot of activity has happened".*

Leaders identified lack of needs-responsive data collection as a barrier to effective utilization of data for decision making. A provincial health minister stated that, "*We find that getting population level information is really difficult. And you can get this information, but it's not*

For peer review only - http://bmjopen.bmj.com/site/about/guidelines.xhtml

really formalized - you just hear verbal estimates. So, in terms of planning, there is no common information base that people have that is like the gold standard". In a similar vein, a leader in a prominent healthcare institution noted, *"There are very few hospitals in Pakistan that actually record quality and patient safety data. So, hospital quality level data, the kind that exists in the NHS, or in the US etc. doesn't exist here".* In addition, most participants noted that gender and equity lens have not been widely considered, neither during the collection and analysis of health data, nor in the design of research and data driven initiatives. With regards to gender, a leader at a non-profit organization explained that: *"Disaggregated data by gender is probably a large problem nationally, particularly with development indicators".* Equity is often overlooked in conversations around data, and when considered, the level of commitment is inadequate. A governmental leader mentioned that: *"Unfortunately, I think equity is more a function of conversations with development partners. And it does translate to some commitment, but not the level of commitment that should be the case".*

The digitization and structure of available data is another holdup, as stated by a manager at a non-governmental organization: "*Some organizations don't have data in a format that's easily accessible, because they don't have electronic systems".* Most participants shared the perception that there is a lack of an appropriately designed system to help collate data to its most desired format. This sentiment was shared by another expert in epidemiology at a tertiary care hospital, who noted that, "*The reality was when I came in, I realized that the structure of the data being collected, was not conducive to be pulled out, right, so we couldn't do any research*". Another expert at an NGO noted*, "When I joined X organization, all this information was collected on different platforms, some of this was paper based, some of it was collected with in-house*

For peer review only - http://bmjopen.bmj.com/site/about/guidelines.xhtml

applications. And none of it was necessarily standards-based. And none of these applications were well designed for information sharing".

Data reliability was another aspect highlighted in the interviews. Specifically in the context of data sharing, a leader at an NGO noted, "*You get conflicting information….who is going to verify whether database A is correct or database B is correct".*

A provincial minister also suggested that while data-driven initiatives do exist, there is sometimes lack of clarity regarding the purpose and objectives of successfully collecting data and ultimately the value attached to these efforts among those collecting and managing this information. "*We tried to give the lady health workers an app to update in real time to update the data about the diseases that they are seeing and the pregnant women that they are seeing. We don't have a proper accountability system and so we cannot tell people that this is something important for health policy and interventions and for a database for knowledge".*

Many interviewees felt that where data did exist, there were other obstacles like significant deficits in staffing and the inability to utilize the collected data. A leader in an NGO noted, "*We do not have trained people in the system, even people who have dealt with data for a long time, do not have the analytical skills to make sense of it, draw conclusions, and ask questions. That has been a real challenge".*

Theme 2: There is openness and enthusiasm for sharing data for advancing health, however, multiple barriers hinder this including appropriate regulatory frameworks, platforms for sharing data, inter-operability, and defined win-win scenarios

For peer review only - http://bmjopen.bmj.com/site/about/guidelines.xhtml

Considering a shared vision to improve health outcomes in Pakistan, leaders indicated overall willingness to share data and partner for this common mission, expressing keen interest and stating their openness to proposals and collaborations. Defining win-win scenarios, in terms of shared objectives between entities and learning from each other's areas of expertise would be critical to organizations sharing data, as a government leader observed: *"I think we are open to proposals where, say, we make data in specific areas available. As long as we get something in return - if I can get more immediate impact out of that process, then that would excite me more. So, you name an area where we can problem-solve, and we can actually close the loop on that partnership (in terms of how we can actually translate it to some impact)".* Interestingly, an epidemiologist at a tertiary care hospital shared insights about ensuring that a shared collaborative keeps 'democracy of data' as a central guiding principle: *"I think there has to be democracy of data sharing within an organization because there's no point hanging on to data, and not sharing it so that somebody can make use of it, and that is one of the problems; people hang on to data as a good treasure that they cannot share with anybody".*

Most experts shared that certain challenges and pertinent questions would need to be accounted for to build a sustainable, shared, accessible data ecosystem. These include the validity and accuracy of datasets themselves, privacy and regulatory framework around data sharing, addressing systemic differences between different sectors and inadequate workforce and training. Experts shared that even if the data exists, dissemination of data is always an issue because data governance is a nascent field in Pakistan. The term governance is also used broadly by interviewees that use it to refer to privacy and security of data as well as an overarching governing structure to establish appropriate ownership of data. An entity leader, at a premier bank, noted that this also holds true for other sectors of Pakistan, such as the well-resourced

For peer review only - http://bmjopen.bmj.com/site/about/guidelines.xhtml

finance sector, where one of the country's largest banks is still working through the early stages of data governance. Furthermore, structuring a data system to be useful requires understanding what data fields one has in their database, what should go into those data fields, and a system that is designed to ensure a clear utilitarian purpose. Though governance appears to be a major barrier for a data collaborative, subjects report that missing-ness and access to data are also impediments to utilization.

A manager and planning executive at a bank, noted: *"Some concerns in data sharing include who will own the data, what will be done with the data? Will the data remain valid or not, will transparency be maintained, privacy rules will be followed or not, ownership of the data? Where will data be used ultimately".*

Similarly, a chief medical officer at a tertiary hospital mentioned that: *"There needs to be a lot more structure put into data sharing, and by structure, what I mean is that rules and regulations (which need to be set up a priori). That will really give confidence to individual institutions and individuals who own that data - that their data is going to be used properly, reliably and honestly".*

Interoperability of data sets was reported as a big challenge due to differences in dataset formats and different data capacity/skills across different institutions and across public and private institutions. Issues of interoperability are further pronounced when complemented with differences in the approach of private vs public sectors and inpatient vs outpatient data. A leader in healthcare administration while describing the public sector healthcare stated that *"Record keeping is something that is very poor there. In-patient record keeping is there, but there is nothing for out-patients at all. The private sector does keep the record, but the public sector does not. But the private sector does not share that data at all".*

For peer review only - http://bmjopen.bmj.com/site/about/guidelines.xhtml

Theme 3: There is limited capacity in the area of both human capital and infrastructure, for being able to use data to advance health but there is appetite to improve and invest in capacity in this area

Inadequate human resources in data management and analytical skills in organizations was identified as a major barrier to both effective internal use of data and external collaborative data sharing efforts. Participants remarked that data sharing through a collaborative, would require capacity-building in this area.

A manager at an NGO noted: *"The issue is not whether people are willing to share data. But certain organizations, traditional non-profit ones for example, don't have data teams or data managers (because of cost budgetary constraints). So, I think that these organizations often don't have the capacity to manage data"*. It was also mentioned that lack of capacity building was a barrier to successfully analyzing and synthesizing data, where many healthcare facilities at the district level were still using a paper-based format for recording data since their staff were not proficient in the use of technology. A provincial healthcare leader noted: *"The main problem here is that we don't have an HR [human resource] there or a proper computerized system there to log in that data and upload it. We have a lot of restrictions in the IT department"*. Similarly, a senior leader at a tertiary care hospital mentioned: "*We have the data, but we don't have the capacity and capability to analyze it and make changes in healthcare"*. There was a clear appetite and keen interest in investing organizational data capacity ranging from investing in needs-responsive data repositories and electronic systems, to upskilling the current workforce. A

For peer review only

For peer review only - http://bmjopen.bmj.com/site/about/guidelines.xhtml

representative from academia shared their experience of setting up a data repository and its potential future impact: *"The long-term objective of this Higher Education Data Repository initiative is that we collect all the granular information throughout a student's life cycle. This will generate a lot of data for analysis, about what kind of educational needs we have in our system, what kind of courses do we need to specialize in for our students and the areas that our faculty members need to specialize in".*

A representative from a large public sector tertiary care hospital reported their initiative of data automation and the barriers associated with it, notably, administrative and financial challenges around staffing plans: *" We have begun an initiative at our hospital, where we are starting to do automation of the data and that is happening but I really wouldn't be able to say to what extent we have been successful with that. The data is within the automation center with the HR. That is also not working because our staffing there has been rejected".* This highlights the multifaceted nature of this dilemma – while some initiatives may be headed in the right direction, approvals to enact and sustain those initiatives are met with challenges.

An NGO leader described the process, components, and importance of building a data-driven team – a cohesive unit of trained individuals that understand the application of data sciences to health. He stated: "*There are four skills that I think are important in building out a data team that we found. One is data engineering, which is just someone who can query databases, particularly complex databases...Then, I think the next skill that we found useful is analytics, which is how to develop dashboards. And that is a very easily trainable skill. So, the third skill that's (commonly) not there is knowing what dashboard to make. And the fourth skill is data*

For peer review only - http://bmjopen.bmj.com/site/about/guidelines.xhtml

science, which is basically being able to model data and that's an even rarer skill especially locally".

A key skill within building capacity to manage and analyze data is the ability to effectively communicate results. A government health leader also delineated the importance of communication in building health data capacity which may be an essential, yet neglected, skill. After data collection and analysis, the success of any subsequent policy and prevention measures depended largely on how they are communicated to people. She stated that, "*There must be a trigger factor that allows the person to do a communications strategy and awareness policy to send that message across. I think communication is very important. You specifically need to know how to communicate".*

A Government leader also stated the need for and importance of on-the-job training by stating that "*For me, the single silver bullet is creating data management routines that act as on the job training for managers".* He added, *"... you need a data boot camp for health leaders, like that is weeklong. And that is sort of morning to night. And that's trying to basically break their thinking and get them to use differently the information that is available, because there is still a lot more information that is available".*

Discussion

Our study is the first, multi-disciplinary endeavor to understand perceptions on health data and health data science in Pakistan. The main finding from our qualitative analysis is that the scope of data science in health for advancing health outcomes, is far-reaching in Pakistan and likely in other LMICs where organizations have collected a great deal of data but are in the early stages of

For peer review only - http://bmjopen.bmj.com/site/about/guidelines.xhtml

understanding how best to leverage and utilize this data. Furthermore, there is potential for establishing a health data ecosystem comprised of a health data collaborative with an appropriate governance structure, intentionality toward data design elements focusing on gender, equity, and needs-responsiveness, that is supported by appropriate capacity building initiatives. Our study findings suggest that while we are at nascent stages of using data to progress a national health agenda, several independent and national efforts are being made to allow for digitization and automation in healthcare and there is keen interest in investment in building capacity in this area. Even though the far-reaching scope of health data and data science methods in healthcare and their potential benefits are recognized by developing countries, development of a national data collaborative that might serve as a foundational block of a larger health data ecosystem is a complex endeavor and presents some challenges.[10] A principal lever for this agenda is timely access to the right information, but this has been a scarce resource in LMICs.[17]

Lack of rigorous and structured systems, problems with accuracy, credibility and completeness, inadequacy of trained personnel with core competencies, and unavailability of analytic tools were core obstacles highlighted by experts. Furthermore, there are very limited efforts to propagate a multimodal, multisectoral, and multidisciplinary approach to data, leading to a conspicuous lack of a central repository of information in LMICs like Pakistan.[8] In line with recent literature, a key concern highlighted by participants in data sharing was safeguarding their privacy, confidentiality, and security, with all interviewees agreeing to the need of a governance and regulatory framework being set up a priori to ensure data transparency and maintaining the trust of all parties involved that their data will be used honestly and reliably.[8,18]

Our participants also stated that the analysis, design, and collection of health data does not currently support gender and equity lens as the core of any organization. Disaggregation of data

For peer review only - http://bmjopen.bmj.com/site/about/guidelines.xhtml

by gender is a problem nationally and is difficult to find. Literature suggests that health system policy development does not always pay adequate attention to gender and even when policies do include gender, intentions can evaporate when it comes to actual implementation.[19,20] Study participants suggested that equity was often a function of conversation with development partners and that the level of commitment to inclusion of equity needed to be increased. Tannenbaum et al. and The World Bank note that gender data is a powerful tool for improving lives as lack of disaggregated gender information has resulted in an incomplete disease understanding.[21,22] Furthermore, gender equity is an integral component of social responsibility and according to International Organization for Standardization (ISO) 26000 Sustainable Development Goal 5 (Guidance on Social Responsibility), whereby the standard denotes the importance of having gender-inclusive leadership and governance in ensuring elimination of gender bias and promotion of gender parity.[23]

Our study participants mentioned how organizations collect and store data, have information management systems, but that data is not being utilized in the most effective way, due to limited capacity and skillsets. [24] Hence, they emphasized that translation and evidence synthesis require significant capacity building. A systematic review reflects on the importance of ongoing training and multilevel strategies needed in development of such programs, and how capacity building can influence different levels of entire organizations and systems.[25] The types of interventions assessed included internet-based teaching and workshops. The results of a worldwide cross-sectional survey by Kaggle et. al, illustrates the extent to which companies in various countries have adapted to machine learning models, with Israel surpassing even the United States.[26] This need also represents an opportunity to develop local, contextual health

For peer review only - http://bmjopen.bmj.com/site/about/guidelines.xhtml

data science programs that equip individuals with appropriate data management and data analytics skills. [27]

A national strategy on establishing a robust health data ecosystem and data collaborative for Pakistan will be an important next step. This necessitates that the gaps identified globally and in our qualitative interviews are bridged and data are put into action. In this regard, a national health digital framework has recently been developed by the Ministry of Health, which can be used for developing a high-level roadmap. As noted by healthcare experts, the roadmap is to help healthcare professionals use data science principles to inform decision making, uplifting research, and guiding clinical approaches to improve healthcare delivery.[28,29]

This study has a few limitations. Our findings mainly stemming from interviews among leaders in the healthcare system of Pakistan do not provide such larger room for generalizability beyond the Global South. However, we present perspectives from a low resource setting which has contextual relevancy and implications for other LMICs in the region. Qualitative interviews focused on perspectives from key management leads at major institutions. This was primarily because the scope and objectives of this exercise were to assimilate input from experts and leaders in management, policy, and healthcare. A next step, on establishing a health data collaborative, will be to ensure data and perspectives from patients and communities, who serve as key stakeholders in healthcare systems.

Conclusion

The present study highlights important opportunities and barriers that need to be addressed to develop a health data ecosystem in Pakistan. Creation of appropriate governance, regulatory frameworks, gender, and equity indicators, and defining win-win scenarios, are important

For peer review only - http://bmjopen.bmj.com/site/about/guidelines.xhtml

principles to consider for planning any national health data collaboratives. To enable this ecosystem, collaboration is required on strategic outlining of how data can be collated, organized, curated, updated, and finally pipelined. For achieving this goal, building data science capacity within organizations would be critical, thus providing the ability to leverage health data to its full potential for informed decision making.

Contributors

All authors confirm that they had full access to all the data in the study and accept responsibility to submit for publication.

ZS conceived of, designed the study, collected data, and acquired funding.

SM collected the data and wrote the original draft of the manuscript.

AAN analyzed and interpreted the data and wrote the original draft of the manuscript.

AM analyzed and interpreted the data and wrote the original draft of the manuscript.

NA wrote the original draft of the manuscript.

SA was involved in data curation and manuscript writing.

JQB was involved in data curation and manuscript writing.

SS wrote the original draft of the manuscript.

ZH wrote the original draft of the manuscript.

ZAB wrote the original draft of the manuscript.

SSV wrote the original draft of the manuscript.

All authors contributed to critically reviewing and editing the manuscript.

Declaration of interests

For peer review only

For peer review only - http://bmjopen.bmj.com/site/about/guidelines.xhtml

Dr Zainab Samad reports research grant support from the NIH-Fogarty International Center for the AKUPI-NCDs Research training program (Project Number 5D43TW011625-02) and UZIMA DS (U54TW012089); the NIHR, UK for the Centre for IMPACT (NIHR203248); Bill & Melinda Gates Foundation (INV 021944 & INV 050389); and Duke University.

Dr Salim S. Virani MD, PhD, FACC, FAHA, FASPC Research support: U.S. Department of Veterans Affairs, National Institutes of Health, World Heart Federation, Tahir and Jooma Family; Honorarium: American College of Cardiology American College of Cardiology (Associate Editor for Innovations, acc.org); Associate Editor (Current Atherosclerosis Reports, Current Cardiology reports, Journal of Clinical Lipidology)

Dr Zulfiqar Bhutta MBBS, FRCPCH, FAAP, PhD Executive Director of NCD Child

Funding

This work was supported through a grant from the Bill & Melinda Gates Foundation (grant number: INV-021944).

Data availability statement

No data are available.

Ethics approval

The study received approval from the Ethical Review Committee at AKU (ERC # 2021-5839-16883).

License statement

For peer review only - http://bmjopen.bmj.com/site/about/guidelines.xhtml

I, the Submitting Author has the right to grant and does grant on behalf of all authors of the Work (as defined in the below author license), an exclusive license and/or a non-exclusive license for contributions from authors who are: i) UK Crown employees; ii) where BMJ has agreed a CC-BY license shall apply, and/or iii) in accordance with the terms applicable for US Federal Government officers or employees acting as part of their official duties; on a worldwide, perpetual, irrevocable, royalty-free basis to BMJ Publishing Group Ltd ("BMJ") its licensees and where the relevant Journal is co-owned by BMJ to the co-owners of the Journal, to publish the Work in BMJ Open and any other BMJ products and to exploit all rights, as set out in our license.

The Submitting Author accepts and understands that any supply made under these terms is made by BMJ to the Submitting Author unless you are acting as an employee on behalf of your employer or a postgraduate student of an affiliated institution which is paying any applicable article publishing charge ("APC") for Open Access articles. Where the Submitting Author wishes to make the Work available on an Open Access basis (and intends to pay the relevant APC), the terms of reuse of such Open Access shall be governed by a Creative Commons license – details of these licenses and which Creative Commons license will apply to this Work are set out in our license referred to above.

Figure legend

Figure 1: An overview of the methodological framework of the study – participant cohorts, process of interview preparation, conductance, and analysis

For peer review only - http://bmjopen.bmj.com/site/about/guidelines.xhtml

References

1 Measuring progress from 1990 to 2017 and projecting attainment to 2030 of the health-related Sustainable Development Goals for 195 countries and territories: a systematic analysis for the Global Burden of Disease Study 2017. *Lancet (London, England)* 2018;**392**:2091–138. doi:10.1016/S0140-6736(18)32281-5

2 Transforming our world: the 2030 Agenda for Sustainable Development | Department of Economic and Social Affairs. https://sdgs.un.org/2030agenda (accessed 21 Nov 2022).

3 Sachs JD, Schmidt-Traub G, Mazzucato M, *et al.* Six Transformations to achieve the Sustainable Development Goals. *Nat Sustain 2019 29* 2019;**2**:805–14. doi:10.1038/s41893-019-0352-9

4 Bhavnani SP, Muñoz D, Bagai A. Data Science in Healthcare: Implications for Early Career Investigators. *Circ Cardiovasc Qual Outcomes* 2016;**9**:683–7. doi:10.1161/CIRCOUTCOMES.116.003081

5 Sharma A, Harrington RA, McClellan MB, *et al.* Using Digital Health Technology to Better Generate Evidence and Deliver Evidence-Based Care. *J Am Coll Cardiol* 2018;**71**:2680–90. doi:10.1016/j.jacc.2018.03.523

6 Ting DSW, Carin L, Dzau V, *et al.* Digital technology and COVID-19. *Nat Med 2020 264*

For peer review only - http://bmjopen.bmj.com/site/about/guidelines.xhtml

2020;**26**:459–61. doi:10.1038/s41591-020-0824-5

7 Imoto S, Hasegawa T, Yamaguchi R. Data science and precision health care. *Nutr Rev* 2020;**78**:53–7. doi:10.1093/nutrit/nuaa110

8 Bezuidenhout L, Chakauya E. Hidden concerns of sharing research data by low/middle-income country scientists. *Glob Bioeth = Probl di Bioet* 2018;**29**:39–54. doi:10.1080/11287462.2018.1441780

9 Wyber R, Vaillancourt S, Perry W, *et al.* Big data in global health: improving health in low- and middle-income countries. *Bull World Health Organ* 2015;**93**:203–8. doi:10.2471/BLT.14.139022

10 Naseem M, Akhund R, Arshad H, *et al.* Exploring the Potential of Artificial Intelligence and Machine Learning to Combat COVID-19 and Existing Opportunities for LMIC: A Scoping Review. *J Prim Care Community Health* 2020;**11**:2150132720963634. doi:10.1177/2150132720963634

11 Brief on Census -2017 | Pakistan Bureau of Statistics. https://www.pbs.gov.pk/content/brief-census-2017 (accessed 21 Nov 2022).

12 Roth GA, Abate D, Abate KH, *et al.* Global, regional, and national age-sex-specific mortality for 282 causes of death in 195 countries and territories, 1980–2017: a systematic analysis for the Global Burden of Disease Study 2017. *Lancet* 2018;**392**:1736–88.http://www.thelancet.com/article/S0140673618322037/fulltext (accessed 21 Nov 2022).

13 Akhtar H, Afridi M, Akhtar S, *et al.* Pakistan's Response to COVID-19: Overcoming National and International Hypes to Fight the Pandemic. *JMIR Public Heal Surveill* 2021;**7**. doi:10.2196/28517

For peer review only

For peer review only - http://bmjopen.bmj.com/site/about/guidelines.xhtml

14 Haq IU, Rehman ZU. Medical Research in Pakistan; A Bibliometric Evaluation from 2001 to 2020. *Libr Philos Pract (e-journal*https://www.pmc.gov.pk/ (accessed 9 Dec 2022).

15 THE 17 GOALS | Sustainable Development. https://sdgs.un.org/goals (accessed 5 Jul 2023).

16 Braun V, Clarke V. Thematic analysis. *APA Handb Res methods Psychol Vol 2 Res Des Quant Qual Neuropsychol Biol* 2012;:57–71. doi:10.1037/13620-004

17 Harrison K, Rahimi N, Danovaro-Holliday MC. Factors limiting data quality in the expanded programme on immunization in low and middle-income countries: A scoping review. *Vaccine* 2020;**38**:4652–63. doi:10.1016/j.vaccine.2020.02.091

18 Tiffin N, George A, LeFevre AE. How to use relevant data for maximal benefit with minimal risk: digital health data governance to protect vulnerable populations in low-income and middle-income countries. *BMJ Glob Heal* 2019;**4**:e001395. doi:10.1136/bmjgh-2019-001395

19 Morgan R, Ayiasi RM, Barman D, *et al.* Gendered health systems: evidence from low- and middle-income countries. *Heal Res policy Syst* 2018;**16**:58. doi:10.1186/s12961-018-0338-5

20 Theobald S, Morgan R, Hawkins K, *et al.* The importance of gender analysis in research for health systems strengthening. Health Policy Plan. 2017;**32**:v1–3. doi:10.1093/heapol/czx163

21 Tannenbaum C, Ellis RP, Eyssel F, *et al.* Sex and gender analysis improves science and engineering. *Nature* 2019;**575**:137–46. doi:10.1038/s41586-019-1657-6

22 More and Better Gender Data: A Powerful Tool for Improving Lives, The World Bank. 2016.

For peer review only - http://bmjopen.bmj.com/site/about/guidelines.xhtml

23 International Organization for Standardization 26000, Guidance on social responsibility.

24 Artificial Intelligence and Start-Ups in Low- and Middle-Income Countries: Progress, Promises and Perils. 2020.

25 DeCorby-Watson K, Mensah G, Bergeron K, *et al.* Effectiveness of capacity building interventions relevant to public health practice: a systematic review. *BMC Public Health* 2018;**18**:684. doi:10.1186/s12889-018-5591-6

26 Bob Hayes. Machine Learning Adoption Rates Around the World. Bus. Broadw. 2021.

27 Hoodbhoy Z, Chunara R, Waljee A, *et al.* Is there a need for graduate-level programmes in health data science? A perspective from Pakistan. *Lancet Glob Heal* 2023;**11**:e23–5. doi:10.1016/S2214-109X(22)00459-4

28 Bates DW, Saria S, Ohno-Machado L, *et al.* Big data in health care: using analytics to identify and manage high-risk and high-cost patients. *Health Aff (Millwood)* 2014;**33**:1123–31. doi:10.1377/hlthaff.2014.0041

29 Shaoibi A, Neelon B, Lenert LA. Shared Decision Making: From Decision Science to Data Science. *Med Decis Mak an Int J Soc Med Decis Mak* 2020;**40**:254–65. doi:10.1177/0272989X20903267

For peer review only - http://bmjopen.bmj.com/site/about/guidelines.xhtml

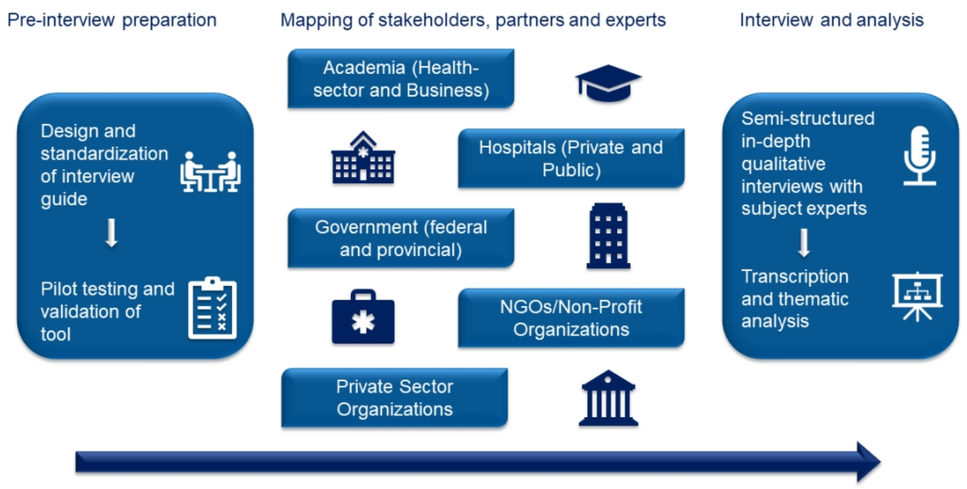

An overview of the methodological framework of the study – participant cohorts, process of interview preparation, conductance, and analysis

108x60mm (300 x 300 DPI)

Semi-structured interview guide

<u>Section 1 : Understanding the health data landscape for Pakistan</u>

What type of health data exists in Pakistan?

Potential prompts in case of a brief reply

- What type of data at a national/regional/global level supports your decision-making ability/research work?
- What type of health data would further support your ability to make informed decisions?
- Is health data at a Pakistan level accessible?
- Is health data at a Pakistan level of good quality? (define quality)

<u>Section 2: Understanding the application of a gender and equity lens to data</u>

Potential prompts in case of a brief reply (mostly guided by the interviewee's response)

- Do we know how to apply a gender/equity lens to our data (disaggregation, analysis etc)
- What population group do you not frequently see available data about?

<u>Section 3: Understanding the organizational handle on health data and its current role</u>

Potential prompts in case of a brief reply (mostly guided by the interviewee's response)

- What health data does your organization hold and to what level does your organization engage with the data for decision making.
- How equipped are organizations to manage the health data they hold?
- What kind of infrastructure/software does your organization have? Is it sufficient?

<u>Section 4: Understanding perceptions around developing a health data science training program/curriculum</u>

How effective do you think the introduction of a health data science training curriculum will be, to address barriers?

Potential prompts in case of a brief reply (mostly guided by the interviewee's response)

- What type of training in data science would be most beneficial to you and why?
- What health data science curriculum/training programs exist and are useful?
- Do you think there's an existing need for development of such a program in Pakistan? Why or why not?
- What barriers should such a training program address?
- What components should the health data science training curriculum have?

For peer review only

Consolidated criteria for reporting qualitative studies (COREQ): 32-item checklist

No. Item	Guide questions/description	Reported on Page #
**Domain 1: Research team and reflexivity**		
Personal Characteristics		
1. Inter viewer/facilitator	Which author/s conducted the interview or focus group?	7
2. Credentials	What were the researcher's credentials? E.g., PhD, MD	8
3. Occupation	What was their occupation at the time of the study?	8
4. Gender	Was the researcher male or female?	7
5. Experience and training	What experience or training did the researcher have?	8
Relationship with participants		
6. Relationship established	There was no personal relationship between interviewers	N/A
7. Participant knowledge of the interviewer	What did the participants know about the researcher? e.g., personal goals, reasons for doing the research	N/A
8. Interviewer characteristics	What characteristics were reported about the inter viewer/facilitator? e.g., Bias, assumptions, reasons and interests in the research topic	N/A

**Domain 2: study design**		
Theoretical framework		

For peer review only - http://bmjopen.bmj.com/site/about/guidelines.xhtml

9. Methodological orientation and Theory	What methodological orientation was stated to underpin the study? e.g., grounded theory, discourse analysis, ethnography, phenomenology, content analysis	9
Participant selection		
10. Sampling	How were participants selected? e.g., purposive, convenience, consecutive, snowball	8
11. Method of approach	How were participants approached? e.g., face-to-face, telephone, mail, email	8
12. Sample size	How many participants were in the study?	10
13. Non-participation	How many people refused to participate or dropped out? Reasons?	N/A
Setting		
14. Setting of data collection	Where was the data collected? e.g., home, clinic, workplace	8
15. Presence of non-participants	Was anyone else present besides the participants and researchers?	8
16. Description of sample	What are the important characteristics of the sample? e.g., demographic data, date	10
Data collection		
17. Interview guide	Were questions, prompts, guides provided by the authors? Was it pilot tested?	7
18. Repeat interviews	Were repeat inter views carried out? If yes, how many?	N/A
19. Audio/visual recording	Did the research use audio or visual recording to collect the data?	9

For peer review only

For peer review only - http://bmjopen.bmj.com/site/about/guidelines.xhtml

20. Field notes	Were field notes made during and/or after the interview or focus group?	N/A
21. Duration	What was the duration of the inter views or focus group?	8
22. Data saturation	Was data saturation discussed?	8
23. Transcripts returned	Were transcripts returned to participants for comment and/or correction?	N/A
**Domain 3: analysis and findings**		
Data analysis		
24. Number of data coders	How many data coders coded the data?	9
25. Description of the coding tree	Did authors provide a description of the coding tree?	N/A
26. Derivation of themes	Were themes identified in advance or derived from the data?	9
27. Software	What software, if applicable, was used to manage the data?	9
28. Participant checking	Did participants provide feedback on the findings?	N/A
Reporting		
29. Quotations presented	Were participant quotations presented to illustrate the themes/findings? Was each quotation identified? e.g., participant number	11-17
30. Data and findings consistent	Was there consistency between the data presented and the findings?	10-17
31. Clarity of major themes	Were major themes clearly presented in the findings?	10-17
32. Clarity of minor themes	Is there a description of diverse cases or discussion of minor themes?	N/A

For peer review only

For peer review only - http://bmjopen.bmj.com/site/about/guidelines.xhtml

For peer review only

For peer review only - http://bmjopen.bmj.com/site/about/guidelines.xhtml

BMJ Open

Health Data Ecosystem in Pakistan – A Multi-sectoral Qualitative Assessment of Needs and Opportunities

Journal:	*BMJ Open*
Manuscript ID	bmjopen-2023-071616.R3
Article Type:	Original research
Date Submitted by the Author:	24-Aug-2023
Complete List of Authors:	Mahmood, Sana; Aga Khan University; Aga Khan University, Department of Community Health Sciences<br>Noorali, Ali Aahil; The Aga Khan University<br>Manji, Afshan; Aga Khan University, Department of Medicine; Aga Khan University<br>Afzal, Noreen; The Aga Khan University<br>Abbas, Saadia; Aga Khan University, Department of Medicine<br>Qamar, Javeria Bilal; Aga Khan University, Department of Medicine<br>Siddiqi, Sameen; Aga Khan University Medical College Pakistan, Community Health Sciences Department<br>Hoodbhoy, Zahra; Aga Khan University, Department of Pediatrics and Child Health<br>Virani, Salim; Michael E. DeBakey Veterans Affairs Medical Center HSR&D, Health Policy, Quality & Informatics Program,<br>Bhutta, Zulfiqar; Hosp Sick Children, Centre for Global Child Health<br>Samad, Zainab; The Aga Khan University; Duke Clinical Research Institute, Department of Medicine
**Primary Subject Heading**:	Public health
Secondary Subject Heading:	Qualitative research
Keywords:	QUALITATIVE RESEARCH, PUBLIC HEALTH, Quality in health care < HEALTH SERVICES ADMINISTRATION & MANAGEMENT

SCHOLARONE™
Manuscripts

I, the Submitting Author has the right to grant and does grant on behalf of all authors of the Work (as defined in the below author licence), an exclusive licence and/or a non-exclusive licence for contributions from authors who are: i) UK Crown employees; ii) where BMJ has agreed a CC-BY licence shall apply, and/or iii) in accordance with the terms applicable for US Federal Government officers or employees acting as part of their official duties; on a worldwide, perpetual, irrevocable, royalty-free basis to BMJ Publishing Group Ltd ("BMJ") its licensees and where the relevant Journal is co-owned by BMJ to the co-owners of the Journal, to publish the Work in this journal and any other BMJ products and to exploit all rights, as set out in our licence.

The Submitting Author accepts and understands that any supply made under these terms is made by BMJ to the Submitting Author unless you are acting as an employee on behalf of your employer or a postgraduate student of an affiliated institution which is paying any applicable article publishing charge ("APC") for Open Access articles. Where the Submitting Author wishes to make the Work available on an Open Access basis (and intends to pay the relevant APC), the terms of reuse of such Open Access shall be governed by a Creative Commons licence – details of these licences and which Creative Commons licence will apply to this Work are set out in our licence referred to above.

Other than as permitted in any relevant BMJ Author's Self Archiving Policies, I confirm this Work has not been accepted for publication elsewhere, is not being considered for publication elsewhere and does not duplicate material already published. I confirm all authors consent to publication of this Work and authorise the granting of this licence.

For peer review only

Health Data Ecosystem in Pakistan – A Multi-sectoral Qualitative Assessment of Needs and Opportunities

Authors: Sana Mahmood EdM [1,2,3], Ali Aahil Noorali MBBS [4,5], Afshan Manji MSc [4,5], Noreen Afzal MPhil [1], Saadia Abbas [4], Javeria Bilal Qamar [4], Sameen Siddiqi DrMed [2], Zahra Hoodbhoy PhD[6], Salim S. Virani PhD[7], Zulfiqar A. Bhutta, PhD [3], Zainab Samad MHS [3,4,8]

Affiliations:

[1] Dean's Office, Medical College, Aga Khan University, Karachi, Pakistan

[2] Department of Community Health Sciences, Aga Khan University, Karachi, Pakistan

[3] Institute of Global Health and Development, Aga Khan University, Karachi, Pakistan

[4] Department of Medicine, Medical College, Aga Khan University, Karachi, Pakistan

[5] CITRIC Health Data Science Center, Medical College, Aga Khan University, Karachi, Pakistan

[6] Department of Pediatrics and Child Health, Aga Khan University, Karachi, Pakistan

[7] Division of Cardiology, Department of Medicine, Baylor College of Medicine, USA

[8] Division of Cardiology, Department of Medicine, Duke University, Duke Global Health Institute, Duke Clinical Research Institute, Durham, NC

Corresponding Author:

Zainab Samad, MBBS, MHS

Aga Khan University, Stadium Road, Karachi, Pakistan, 74800

Email: samad.zainab@aku.edu

Phone: 03002017196

For peer review only - http://bmjopen.bmj.com/site/about/guidelines.xhtml

Word count:

4,409 words

For peer review only

For peer review only - http://bmjopen.bmj.com/site/about/guidelines.xhtml

Abstract

Objective:

Data are essential for tracking and monitoring of progress on health-related sustainable development goals (SDGs). But the capacity to analyze subnational and granular data is limited in low- and middle-income countries. Although Pakistan lags behind on achieving several health-related SDGs, its health information capacity is nascent. Through an exploratory qualitative approach, we aimed to understand the current landscape and perceptions on data in decision making among stakeholders of the health data ecosystem in Pakistan.

Design:

We used an exploratory qualitative study design.

Setting:

This study was conducted at the Aga Khan University, Karachi, Pakistan.

Participants:

We conducted semi-structured, in-depth interviews with multidisciplinary and multisectoral stakeholders from academia, hospital management, government, NGOs, and other relevant private entities till thematic saturation was achieved. Interviews were recorded and transcribed, followed by thematic analysis using NVivo.

Results:

Thematic analysis of 15 in-depth interviews revealed three major themes: 1) Institutions are collecting data, but face barriers to its effective utilization for decision making. These include lack of collection of needs-responsive data, lack of a gender/equity in data collection efforts, inadequate digitization, data reliability, and limited analytical ability; 2) There is openness and enthusiasm for sharing data for advancing health, however, multiple barriers hinder this

For peer review only

including appropriate regulatory frameworks, platforms for sharing data, inter-operability, and defined win-win scenarios; 3) There is limited capacity in the area of both human capital and infrastructure, for being able to use data to advance health but there is appetite to improve and invest in capacity in this area

Conclusions:

Our study identified key areas of focus that can contribute to orient a national health data roadmap and ecosystem in Pakistan.

For peer review only

For peer review only - http://bmjopen.bmj.com/site/about/guidelines.xhtml

Strengths and limitations of this study

- Our study participants were experts and decision makers from multiple sectors, across provinces, and with work at the intersection of health and data science.
- In-depth interviews with key-informants allowed for a thorough exploration of the scope and challenges for health data science in Pakistan.
- We did not conduct patient interviews to learn about their opinions about the application of health data science in Pakistan.

For peer review only

Introduction

Data are essential for tracking and monitoring progress on health-related sustainable development goals (SDGs).[1–3] While data and data analytics are being used in high income countries (HICs) to improve health equity, health outcomes, and continuously inform healthcare systems, their use in low-middle-income countries (LMICs) is lagging.[4–8] Investing in data ecosystems represents an important opportunity for monitoring and quickening progress on health-related SDGs in LMICs.[1,8–10]

With a population of 230 million, Pakistan, the fifth most populous LMIC, has a high estimated mortality and morbidity burden for various diseases, but its health system and health information system capacity is nascent.[11,12] However, during the COVID-19 pandemic, data were made nationally available in almost real-time, and data science methods were used to inform health policy and population level interventions such as smart lock downs and vaccinations efforts.[13,14] Multi-stakeholder and interprovincial collaboration underpinned this successful effort and highlighted the need for a national health data ecosystem outside of crisis situations. To inform such future efforts, an understanding of the current perceptions around health data, its use in decision making and the health data ecosystem in Pakistan is required. To this end, we adopted a qualitative approach to understand the current landscape as well as perceptions on data in decision-making among a wide range of stakeholders.

For peer review only

For peer review only - http://bmjopen.bmj.com/site/about/guidelines.xhtml

Methods

Study Design and Setting:

This was an exploratory qualitative study with the primary objectives to comprehend the scope of the health data ecosystem in Pakistan, knowledge and attitudes around developing partnerships and sharing data, and perceptions around the need for developing health data science capacity in Pakistan.

The study was led by investigators at the Aga Khan University (AKU) in Pakistan. With a forty-year presence in Pakistan, AKU has well established partnerships at both provincial and national levels, with government and academia, enabling regular engagement in interdisciplinary policy discussions and fora.

Study instrument:

A semi-structured interview guide was designed using carefully curated questions (available in supplement 1). The guide prompted a detailed discussion on the landscape and scope of existing health data. Further discussion was rooted in potential facilitators and barriers to building a national health data collaborative that would contribute to improved health outcomes in Pakistan. This included understanding the nature of existing policies and collaboratives, the availability and need of human capital for health data initiatives, and structures—from governance to infrastructure, which were present or would need to be developed and implemented to allow for organizations across sectors to comfortably share data to advance health outcomes in Pakistan.

The guide was pilot-tested among a diverse cohort of four individuals and judged for clarity of questions. Feedback from the pilot testing was incorporated to address gaps in the interview

For peer review only - http://bmjopen.bmj.com/site/about/guidelines.xhtml

guide. Interviews were conducted by two female investigators (ZS and SM), who were the departmental chair and director, respectively, while the research staff (AAN, AA, SA, JBQ) acted as observers. Standardization was maintained across all interviews by ensuring that the same two interviewers conducted all the interviews with the same guide. Both interviewers had prior experience of conducting qualitative interviews. Each interview was conducted online for a duration that varied between 30 minutes and 2 hours.

Sampling, Inclusion and Exclusion Criteria:

A scoping exercise was conducted to identify experts and relevant institutions. Through discussion, the investigators collectively identified key sectors in the health ecosystem of Pakistan for a landscape analysis which formed the inclusion criteria: 1. University and academia with faculty in health and/or information technology (IT), 2. Senior-level hospital management (both private and public) 3. Government ministers (federal and provincial/state), 4. Non-governmental organizations (NGOs) and 5. Private-sector organizations (pharmaceutical, finance, and health insurance sectors). There were no major or minor exclusion criteria.

Based on the SDGs 4, 5 and 9 [15] (quality education, gender equality and industry, innovation and infrastructure respectively), we performed a mapping of major institutions across these domains in the public and private health sectors, private organizations and NGOs. Major institutions were defined as those that were expected to have organizational maturity and scale in the area and capacity to collect data. Lastly, individuals with at least five years of leadership experience in their respective domains were eligible to participate in the study.

For peer review only - http://bmjopen.bmj.com/site/about/guidelines.xhtml

Following convenience sampling to select key stakeholders with a particular focus on those with management/decision-making roles, invites were extended via email, and interviews were arranged. Thematic saturation was reached at 15 interviews which comprised the final study sample. Figure 1 illustrates the study methodology.

Data Analysis:

The six-step method of thematic analysis of Braun and Clarke (2006) guided the analytical process.[16] Interviews were first audio recorded and transcribed verbatim. Since the research staff were not only observers, but also transcribers for the interviews, these roles helped them get familiarized and immersed with the collected data. Transcripts were imported into NVivo (version 12). An initial list of a priori codes was used by two research team members (AAN and AM) to code the transcripts. New codes emerging from the data, which were deemed relevant to the study objectives were also added to this list. This codebook was refined through iteration and consensus among research staff which helped in standardization of the codes applied in all transcripts.

Data coded under similar codes were then grouped to identify major themes, which were paired with direct verbatim quotations from the interviewees. The themes were then reviewed to ensure adequate data and participant quotations supported the creation of each theme. All themes were then defined to convey an adequate description of its subthemes and relevant data. Lastly, the results were written in a format to describe the analyzed data in detail.

Patient and Public Involvement

For peer review only - http://bmjopen.bmj.com/site/about/guidelines.xhtml

Patients and the public were not involved in the research design, analysis, and dissemination of the findings.

Ethical Considerations:

The study received approval from the Ethical Review Committee at AKU (ERC # 2021-5839-16883). Written informed consent over email for the study was obtained from each participant before starting the interview.

Results

We conducted 15 in-depth interviews with a diverse range of stakeholders from five centralized cohorts. Sector and designation of the participants are described in Table 1. Thematic analysis generated three overarching themes: 1) Institutions are collecting data, but face barriers to its effective utilization for decision making. These include lack of collection of needs-responsive data, lack of a gender/equity in data collection efforts, inadequate digitization, data reliability, and limited analytical ability.; 2) There is openness and enthusiasm for sharing data for advancing health, however, multiple barriers hinder this endeavor; 3) There is limited capacity in the area of both human capital and infrastructure, for being able to use data to advance health but there is appetite to improve capacity in this area.

**Table 1: Sector and designation of study participants (n=15)**	
**Sector**	**N (%)**
Academia	1
Hospitals	2
Government	3
Non-governmental organizations (NGOs)	3
Private-sector organizations	6
**Designation**	

For peer review only - http://bmjopen.bmj.com/site/about/guidelines.xhtml

Mid-level management	5
Chief medical officer	2
Health minister	2
Senior management	6

Theme 1: Institutions are collecting data, but face barriers to its effective utilization for decision making. These include lack of collection of needs-responsive data, lack of gender/equity in data collection efforts, inadequate digitization, data reliability and limited analytical ability.

Experts communicated that there are several initiatives at the intersection of health and data, and organizations are collecting and holding data, but these initiatives exist in silos. Institutions have a large volume of operational data, but several barriers to their effective utilization were identified. These ranged from lack of collection of needs-responsive data (data not capturing variables required for decision making, interventions and policy reform), inadequate digitization, inappropriate data formats, data reliability, value placed on ultimate data use by data collectors, and human capacity to gauge scientific insights from it.

Inadequate data mapping and lack of dataset awareness within institutions affecting access and use were identified as barriers. An expert in digital health strategy noted that *"I would think that the dissemination of the data is a far bigger issue than the data holes being there. So, if you start digging, you find data sets, but you see that no one is aware of them, even though a lot of activity has happened".*

Leaders identified lack of needs-responsive data collection as a barrier to effective utilization of data for decision making. A provincial health minister stated that, "*We find that getting population level information is really difficult. And you can get this information, but it's not*

For peer review only - http://bmjopen.bmj.com/site/about/guidelines.xhtml

really formalized - you just hear verbal estimates. So, in terms of planning, there is no common information base that people have that is like the gold standard". In a similar vein, a leader in a prominent healthcare institution noted, *"There are very few hospitals in Pakistan that actually record quality and patient safety data. So, hospital quality level data, the kind that exists in the NHS, or in the US etc. doesn't exist here".* In addition, most participants noted that gender and equity lens have not been widely considered, neither during the collection and analysis of health data, nor in the design of research and data driven initiatives. With regards to gender, a leader at a non-profit organization explained that: *"Disaggregated data by gender is probably a large problem nationally, particularly with development indicators".* Equity is often overlooked in conversations around data, and when considered, the level of commitment is inadequate. A governmental leader mentioned that: *"Unfortunately, I think equity is more a function of conversations with development partners. And it does translate to some commitment, but not the level of commitment that should be the case".*

The digitization and structure of available data is another holdup, as stated by a manager at a non-governmental organization: "*Some organizations don't have data in a format that's easily accessible, because they don't have electronic systems".* Most participants shared the perception that there is a lack of an appropriately designed system to help collate data to its most desired format. This sentiment was shared by another expert in epidemiology at a tertiary care hospital, who noted that, "*The reality was when I came in, I realized that the structure of the data being collected, was not conducive to be pulled out, right, so we couldn't do any research*". Another expert at an NGO noted*, "When I joined X organization, all this information was collected on different platforms, some of this was paper based, some of it was collected with in-house*

For peer review only - http://bmjopen.bmj.com/site/about/guidelines.xhtml

applications. And none of it was necessarily standards-based. And none of these applications were well designed for information sharing".

Data reliability was another aspect highlighted in the interviews. Specifically in the context of data sharing, a leader at an NGO noted, "*You get conflicting information….who is going to verify whether database A is correct or database B is correct".*

A provincial minister also suggested that while data-driven initiatives do exist, there is sometimes lack of clarity regarding the purpose and objectives of successfully collecting data and ultimately the value attached to these efforts among those collecting and managing this information. "*We tried to give the lady health workers an app to update in real time to update the data about the diseases that they are seeing and the pregnant women that they are seeing. We don't have a proper accountability system and so we cannot tell people that this is something important for health policy and interventions and for a database for knowledge".*

Many interviewees felt that where data did exist, there were other obstacles like significant deficits in staffing and the inability to utilize the collected data. A leader in an NGO noted, "*We do not have trained people in the system, even people who have dealt with data for a long time, do not have the analytical skills to make sense of it, draw conclusions, and ask questions. That has been a real challenge".*

Theme 2: There is openness and enthusiasm for sharing data for advancing health, however, multiple barriers hinder this including appropriate regulatory frameworks, platforms for sharing data, inter-operability, and defined win-win scenarios

For peer review only - http://bmjopen.bmj.com/site/about/guidelines.xhtml

Considering a shared vision to improve health outcomes in Pakistan, leaders indicated overall willingness to share data and partner for this common mission, expressing keen interest and stating their openness to proposals and collaborations. Defining win-win scenarios, in terms of shared objectives between entities and learning from each other's areas of expertise would be critical to organizations sharing data, as a government leader observed: *"I think we are open to proposals where, say, we make data in specific areas available. As long as we get something in return - if I can get more immediate impact out of that process, then that would excite me more. So, you name an area where we can problem-solve, and we can actually close the loop on that partnership (in terms of how we can actually translate it to some impact)".* Interestingly, an epidemiologist at a tertiary care hospital shared insights about ensuring that a shared collaborative keeps 'democracy of data' as a central guiding principle: *"I think there has to be democracy of data sharing within an organization because there's no point hanging on to data, and not sharing it so that somebody can make use of it, and that is one of the problems; people hang on to data as a good treasure that they cannot share with anybody".*

Most experts shared that certain challenges and pertinent questions would need to be accounted for to build a sustainable, shared, accessible data ecosystem. These include the validity and accuracy of datasets themselves, privacy and regulatory framework around data sharing, addressing systemic differences between different sectors and inadequate workforce and training. Experts shared that even if the data exists, dissemination of data is always an issue because data governance is a nascent field in Pakistan. The term governance is also used broadly by interviewees that use it to refer to privacy and security of data as well as an overarching governing structure to establish appropriate ownership of data. An entity leader, at a premier bank, noted that this also holds true for other sectors of Pakistan, such as the well-resourced

For peer review only - http://bmjopen.bmj.com/site/about/guidelines.xhtml

finance sector, where one of the country's largest banks is still working through the early stages of data governance. Furthermore, structuring a data system to be useful requires understanding what data fields one has in their database, what should go into those data fields, and a system that is designed to ensure a clear utilitarian purpose. Though governance appears to be a major barrier for a data collaborative, subjects report that missing-ness and access to data are also impediments to utilization.

A manager and planning executive at a bank, noted: *"Some concerns in data sharing include who will own the data, what will be done with the data? Will the data remain valid or not, will transparency be maintained, privacy rules will be followed or not, ownership of the data? Where will data be used ultimately".*

Similarly, a chief medical officer at a tertiary hospital mentioned that: *"There needs to be a lot more structure put into data sharing, and by structure, what I mean is that rules and regulations (which need to be set up a priori). That will really give confidence to individual institutions and individuals who own that data - that their data is going to be used properly, reliably and honestly".*

Interoperability of data sets was reported as a big challenge due to differences in dataset formats and different data capacity/skills across different institutions and across public and private institutions. Issues of interoperability are further pronounced when complemented with differences in the approach of private vs public sectors and inpatient vs outpatient data. A leader in healthcare administration while describing the public sector healthcare stated that *"Record keeping is something that is very poor there. In-patient record keeping is there, but there is nothing for out-patients at all. The private sector does keep the record, but the public sector does not. But the private sector does not share that data at all".*

For peer review only - http://bmjopen.bmj.com/site/about/guidelines.xhtml

For peer review only

Theme 3: There is limited capacity in the area of both human capital and infrastructure, for being able to use data to advance health but there is appetite to improve and invest in capacity in this area

Inadequate human resources in data management and analytical skills in organizations was identified as a major barrier to both effective internal use of data and external collaborative data sharing efforts. Participants remarked that data sharing through a collaborative, would require capacity-building in this area.

A manager at an NGO noted: *"The issue is not whether people are willing to share data. But certain organizations, traditional non-profit ones for example, don't have data teams or data managers (because of cost budgetary constraints). So, I think that these organizations often don't have the capacity to manage data"*. It was also mentioned that lack of capacity building was a barrier to successfully analyzing and synthesizing data, where many healthcare facilities at the district level were still using a paper-based format for recording data since their staff were not proficient in the use of technology. A provincial healthcare leader noted: *"The main problem here is that we don't have an HR [human resource] there or a proper computerized system there to log in that data and upload it. We have a lot of restrictions in the IT department"*. Similarly, a senior leader at a tertiary care hospital mentioned: "*We have the data, but we don't have the capacity and capability to analyze it and make changes in healthcare"*. There was a clear appetite and keen interest in investing organizational data capacity ranging from investing in needs-responsive data repositories and electronic systems, to upskilling the current workforce. A

For peer review only - http://bmjopen.bmj.com/site/about/guidelines.xhtml

representative from academia shared their experience of setting up a data repository and its potential future impact: *"The long-term objective of this Higher Education Data Repository initiative is that we collect all the granular information throughout a student's life cycle. This will generate a lot of data for analysis, about what kind of educational needs we have in our system, what kind of courses do we need to specialize in for our students and the areas that our faculty members need to specialize in".*

A representative from a large public sector tertiary care hospital reported their initiative of data automation and the barriers associated with it, notably, administrative and financial challenges around staffing plans: *" We have begun an initiative at our hospital, where we are starting to do automation of the data and that is happening but I really wouldn't be able to say to what extent we have been successful with that. The data is within the automation center with the HR. That is also not working because our staffing there has been rejected".* This highlights the multifaceted nature of this dilemma – while some initiatives may be headed in the right direction, approvals to enact and sustain those initiatives are met with challenges.

An NGO leader described the process, components, and importance of building a data-driven team – a cohesive unit of trained individuals that understand the application of data sciences to health. He stated: "*There are four skills that I think are important in building out a data team that we found. One is data engineering, which is just someone who can query databases, particularly complex databases...Then, I think the next skill that we found useful is analytics, which is how to develop dashboards. And that is a very easily trainable skill. So, the third skill that's (commonly) not there is knowing what dashboard to make. And the fourth skill is data*

For peer review only - http://bmjopen.bmj.com/site/about/guidelines.xhtml

science, which is basically being able to model data and that's an even rarer skill especially locally".

A key skill within building capacity to manage and analyze data is the ability to effectively communicate results. A government health leader also delineated the importance of communication in building health data capacity which may be an essential, yet neglected, skill. After data collection and analysis, the success of any subsequent policy and prevention measures depended largely on how they are communicated to people. She stated that, "*There must be a trigger factor that allows the person to do a communications strategy and awareness policy to send that message across. I think communication is very important. You specifically need to know how to communicate".*

A Government leader also stated the need for and importance of on-the-job training by stating that "*For me, the single silver bullet is creating data management routines that act as on the job training for managers".* He added, *"... you need a data boot camp for health leaders, like that is weeklong. And that is sort of morning to night. And that's trying to basically break their thinking and get them to use differently the information that is available, because there is still a lot more information that is available".*

Discussion

Our study is the first, multi-disciplinary endeavor to understand perceptions on health data and health data science in Pakistan. The main finding from our qualitative analysis is that the scope of data science in health for advancing health outcomes, is far-reaching in Pakistan and likely in other LMICs where organizations have collected a great deal of data but are in the early stages of

For peer review only - http://bmjopen.bmj.com/site/about/guidelines.xhtml

understanding how best to leverage and utilize this data. Furthermore, there is potential for establishing a health data ecosystem comprised of a health data collaborative with an appropriate governance structure, intentionality toward data design elements focusing on gender, equity, and needs-responsiveness, that is supported by appropriate capacity building initiatives. Our study findings suggest that while we are at nascent stages of using data to progress a national health agenda, several independent and national efforts are being made to allow for digitization and automation in healthcare and there is keen interest in investment in building capacity in this area. Even though the far-reaching scope of health data and data science methods in healthcare and their potential benefits are recognized by developing countries, development of a national data collaborative that might serve as a foundational block of a larger health data ecosystem is a complex endeavor and presents some challenges.[10] A principal lever for this agenda is timely access to the right information, but this has been a scarce resource in LMICs.[17]

Lack of rigorous and structured systems, problems with accuracy, credibility and completeness, inadequacy of trained personnel with core competencies, and unavailability of analytic tools were core obstacles highlighted by experts. Furthermore, there are very limited efforts to propagate a multimodal, multisectoral, and multidisciplinary approach to data, leading to a conspicuous lack of a central repository of information in LMICs like Pakistan.[8] In line with recent literature, a key concern highlighted by participants in data sharing was safeguarding their privacy, confidentiality, and security, with all interviewees agreeing to the need of a governance and regulatory framework being set up a priori to ensure data transparency and maintaining the trust of all parties involved that their data will be used honestly and reliably.[8,18]

Our participants also stated that the analysis, design, and collection of health data does not currently support gender and equity lens as the core of any organization. Disaggregation of data

For peer review only - http://bmjopen.bmj.com/site/about/guidelines.xhtml

by gender is a problem nationally and is difficult to find. Literature suggests that health system policy development does not always pay adequate attention to gender and even when policies do include gender, intentions can evaporate when it comes to actual implementation.[19,20] Study participants suggested that equity was often a function of conversation with development partners and that the level of commitment to inclusion of equity needed to be increased. Tannenbaum et al. and The World Bank note that gender data is a powerful tool for improving lives as lack of disaggregated gender information has resulted in an incomplete disease understanding.[21,22] Furthermore, gender equity is an integral component of social responsibility and according to International Organization for Standardization (ISO) 26000 Sustainable Development Goal 5 (Guidance on Social Responsibility), whereby the standard denotes the importance of having gender-inclusive leadership and governance in ensuring elimination of gender bias and promotion of gender parity.[23]

Our study participants mentioned how organizations collect and store data, have information management systems, but that data is not being utilized in the most effective way, due to limited capacity and skillsets. [24] Hence, they emphasized that translation and evidence synthesis require significant capacity building. A systematic review reflects on the importance of ongoing training and multilevel strategies needed in development of such programs, and how capacity building can influence different levels of entire organizations and systems.[25] The types of interventions assessed included internet-based teaching and workshops. The results of a worldwide cross-sectional survey by Kaggle et. al, illustrates the extent to which companies in various countries have adapted to machine learning models, with Israel surpassing even the United States.[26] This need also represents an opportunity to develop local, contextual health

For peer review only - http://bmjopen.bmj.com/site/about/guidelines.xhtml

data science programs that equip individuals with appropriate data management and data analytics skills. [27]

A national strategy on establishing a robust health data ecosystem and data collaborative for Pakistan will be an important next step. This necessitates that the gaps identified globally and in our qualitative interviews are bridged and data are put into action. In this regard, a national health digital framework has recently been developed by the Ministry of Health, which can be used for developing a high-level roadmap. As noted by healthcare experts, the roadmap is to help healthcare professionals use data science principles to inform decision making, uplifting research, and guiding clinical approaches to improve healthcare delivery.[28,29]

This study has a few limitations. Our findings mainly stemming from interviews among leaders in the healthcare system of Pakistan do not provide such larger room for generalizability beyond the Global South. However, we present perspectives from a low resource setting which has contextual relevancy and implications for other LMICs in the region. Qualitative interviews focused on perspectives from key management leads at major institutions. This was primarily because the scope and objectives of this exercise were to assimilate input from experts and leaders in management, policy, and healthcare. A next step, on establishing a health data collaborative, will be to ensure data and perspectives from patients and communities, who serve as key stakeholders in healthcare systems.

Conclusion

The present study highlights important opportunities and barriers that need to be addressed to develop a health data ecosystem in Pakistan. Creation of appropriate governance, regulatory frameworks, gender, and equity indicators, and defining win-win scenarios, are important

For peer review only - http://bmjopen.bmj.com/site/about/guidelines.xhtml

principles to consider for planning any national health data collaboratives. To enable this ecosystem, collaboration is required on strategic outlining of how data can be collated, organized, curated, updated, and finally pipelined. For achieving this goal, building data science capacity within organizations would be critical, thus providing the ability to leverage health data to its full potential for informed decision making.

Contributors

All authors confirm that they had full access to all the data in the study and accept responsibility to submit for publication.

ZS conceived of, designed the study, collected data, and acquired funding.

SM collected the data and wrote the original draft of the manuscript.

AAN analyzed and interpreted the data and wrote the original draft of the manuscript.

AM analyzed and interpreted the data and wrote the original draft of the manuscript.

NA wrote the original draft of the manuscript.

SA was involved in data curation and manuscript writing.

JQB was involved in data curation and manuscript writing.

SS wrote the original draft of the manuscript.

ZH wrote the original draft of the manuscript.

ZAB wrote the original draft of the manuscript.

SSV wrote the original draft of the manuscript.

All authors contributed to critically reviewing and editing the manuscript.

Declaration of interests

For peer review only

For peer review only - http://bmjopen.bmj.com/site/about/guidelines.xhtml

Dr Zainab Samad reports research grant support from the NIH-Fogarty International Center for the AKUPI-NCDs Research training program (Project Number 5D43TW011625-02) and UZIMA DS (U54TW012089); the NIHR, UK for the Centre for IMPACT (NIHR203248); Bill & Melinda Gates Foundation (INV 021944 & INV 050389); and Duke University.

Dr Salim S. Virani MD, PhD, FACC, FAHA, FASPC Research support: U.S. Department of Veterans Affairs, National Institutes of Health, World Heart Federation, Tahir and Jooma Family; Honorarium: American College of Cardiology American College of Cardiology (Associate Editor for Innovations, acc.org); Associate Editor (Current Atherosclerosis Reports, Current Cardiology reports, Journal of Clinical Lipidology)

Dr Zulfiqar Bhutta MBBS, FRCPCH, FAAP, PhD Executive Director of NCD Child

Funding

This work was supported through a grant from the Bill & Melinda Gates Foundation (grant number: INV-021944).

Data availability statement

No data are available.

Ethics approval

The study received approval from the Ethical Review Committee at AKU (ERC # 2021-5839-16883).

License statement

For peer review only - http://bmjopen.bmj.com/site/about/guidelines.xhtml

I, the Submitting Author has the right to grant and does grant on behalf of all authors of the Work (as defined in the below author license), an exclusive license and/or a non-exclusive license for contributions from authors who are: i) UK Crown employees; ii) where BMJ has agreed a CC-BY license shall apply, and/or iii) in accordance with the terms applicable for US Federal Government officers or employees acting as part of their official duties; on a worldwide, perpetual, irrevocable, royalty-free basis to BMJ Publishing Group Ltd ("BMJ") its licensees and where the relevant Journal is co-owned by BMJ to the co-owners of the Journal, to publish the Work in BMJ Open and any other BMJ products and to exploit all rights, as set out in our license.

The Submitting Author accepts and understands that any supply made under these terms is made by BMJ to the Submitting Author unless you are acting as an employee on behalf of your employer or a postgraduate student of an affiliated institution which is paying any applicable article publishing charge ("APC") for Open Access articles. Where the Submitting Author wishes to make the Work available on an Open Access basis (and intends to pay the relevant APC), the terms of reuse of such Open Access shall be governed by a Creative Commons license – details of these licenses and which Creative Commons license will apply to this Work are set out in our license referred to above.

Figure legend

Figure 1: An overview of the methodological framework of the study – participant cohorts, process of interview preparation, conductance, and analysis

For peer review only - http://bmjopen.bmj.com/site/about/guidelines.xhtml

References

1 Measuring progress from 1990 to 2017 and projecting attainment to 2030 of the health-related Sustainable Development Goals for 195 countries and territories: a systematic analysis for the Global Burden of Disease Study 2017. *Lancet (London, England)* 2018;**392**:2091–138. doi:10.1016/S0140-6736(18)32281-5

2 Transforming our world: the 2030 Agenda for Sustainable Development | Department of Economic and Social Affairs. https://sdgs.un.org/2030agenda (accessed 21 Nov 2022).

3 Sachs JD, Schmidt-Traub G, Mazzucato M, *et al.* Six Transformations to achieve the Sustainable Development Goals. *Nat Sustain 2019 29* 2019;**2**:805–14. doi:10.1038/s41893-019-0352-9

4 Bhavnani SP, Muñoz D, Bagai A. Data Science in Healthcare: Implications for Early Career Investigators. *Circ Cardiovasc Qual Outcomes* 2016;**9**:683–7. doi:10.1161/CIRCOUTCOMES.116.003081

5 Sharma A, Harrington RA, McClellan MB, *et al.* Using Digital Health Technology to Better Generate Evidence and Deliver Evidence-Based Care. *J Am Coll Cardiol* 2018;**71**:2680–90. doi:10.1016/j.jacc.2018.03.523

6 Ting DSW, Carin L, Dzau V, *et al.* Digital technology and COVID-19. *Nat Med 2020 264*

For peer review only - http://bmjopen.bmj.com/site/about/guidelines.xhtml

2020;**26**:459–61. doi:10.1038/s41591-020-0824-5

7 Imoto S, Hasegawa T, Yamaguchi R. Data science and precision health care. *Nutr Rev* 2020;**78**:53–7. doi:10.1093/nutrit/nuaa110

8 Bezuidenhout L, Chakauya E. Hidden concerns of sharing research data by low/middle-income country scientists. *Glob Bioeth = Probl di Bioet* 2018;**29**:39–54. doi:10.1080/11287462.2018.1441780

9 Wyber R, Vaillancourt S, Perry W, *et al.* Big data in global health: improving health in low- and middle-income countries. *Bull World Health Organ* 2015;**93**:203–8. doi:10.2471/BLT.14.139022

10 Naseem M, Akhund R, Arshad H, *et al.* Exploring the Potential of Artificial Intelligence and Machine Learning to Combat COVID-19 and Existing Opportunities for LMIC: A Scoping Review. *J Prim Care Community Health* 2020;**11**:2150132720963634. doi:10.1177/2150132720963634

11 Brief on Census -2017 | Pakistan Bureau of Statistics. https://www.pbs.gov.pk/content/brief-census-2017 (accessed 21 Nov 2022).

12 Roth GA, Abate D, Abate KH, *et al.* Global, regional, and national age-sex-specific mortality for 282 causes of death in 195 countries and territories, 1980–2017: a systematic analysis for the Global Burden of Disease Study 2017. *Lancet* 2018;**392**:1736–88.http://www.thelancet.com/article/S0140673618322037/fulltext (accessed 21 Nov 2022).

13 Akhtar H, Afridi M, Akhtar S, *et al.* Pakistan's Response to COVID-19: Overcoming National and International Hypes to Fight the Pandemic. *JMIR Public Heal Surveill* 2021;**7**. doi:10.2196/28517

For peer review only - http://bmjopen.bmj.com/site/about/guidelines.xhtml

14 Haq IU, Rehman ZU. Medical Research in Pakistan; A Bibliometric Evaluation from 2001 to 2020. *Libr Philos Pract (e-journal*https://www.pmc.gov.pk/ (accessed 9 Dec 2022).

15 THE 17 GOALS | Sustainable Development. https://sdgs.un.org/goals (accessed 5 Jul 2023).

16 Braun V, Clarke V. Thematic analysis. *APA Handb Res methods Psychol Vol 2 Res Des Quant Qual Neuropsychol Biol* 2012;:57–71. doi:10.1037/13620-004

17 Harrison K, Rahimi N, Danovaro-Holliday MC. Factors limiting data quality in the expanded programme on immunization in low and middle-income countries: A scoping review. *Vaccine* 2020;**38**:4652–63. doi:10.1016/j.vaccine.2020.02.091

18 Tiffin N, George A, LeFevre AE. How to use relevant data for maximal benefit with minimal risk: digital health data governance to protect vulnerable populations in low-income and middle-income countries. *BMJ Glob Heal* 2019;**4**:e001395. doi:10.1136/bmjgh-2019-001395

19 Morgan R, Ayiasi RM, Barman D, *et al.* Gendered health systems: evidence from low- and middle-income countries. *Heal Res policy Syst* 2018;**16**:58. doi:10.1186/s12961-018-0338-5

20 Theobald S, Morgan R, Hawkins K, *et al.* The importance of gender analysis in research for health systems strengthening. Health Policy Plan. 2017;**32**:v1–3. doi:10.1093/heapol/czx163

21 Tannenbaum C, Ellis RP, Eyssel F, *et al.* Sex and gender analysis improves science and engineering. *Nature* 2019;**575**:137–46. doi:10.1038/s41586-019-1657-6

22 More and Better Gender Data: A Powerful Tool for Improving Lives, The World Bank. 2016.

For peer review only - http://bmjopen.bmj.com/site/about/guidelines.xhtml

23 International Organization for Standardization 26000, Guidance on social responsibility.

24 Artificial Intelligence and Start-Ups in Low- and Middle-Income Countries: Progress, Promises and Perils. 2020.

25 DeCorby-Watson K, Mensah G, Bergeron K, *et al.* Effectiveness of capacity building interventions relevant to public health practice: a systematic review. *BMC Public Health* 2018;**18**:684. doi:10.1186/s12889-018-5591-6

26 Bob Hayes. Machine Learning Adoption Rates Around the World. Bus. Broadw. 2021.

27 Hoodbhoy Z, Chunara R, Waljee A, *et al.* Is there a need for graduate-level programmes in health data science? A perspective from Pakistan. *Lancet Glob Heal* 2023;**11**:e23–5. doi:10.1016/S2214-109X(22)00459-4

28 Bates DW, Saria S, Ohno-Machado L, *et al.* Big data in health care: using analytics to identify and manage high-risk and high-cost patients. *Health Aff (Millwood)* 2014;**33**:1123–31. doi:10.1377/hlthaff.2014.0041

29 Shaoibi A, Neelon B, Lenert LA. Shared Decision Making: From Decision Science to Data Science. *Med Decis Mak an Int J Soc Med Decis Mak* 2020;**40**:254–65. doi:10.1177/0272989X20903267

For peer review only

For peer review only - http://bmjopen.bmj.com/site/about/guidelines.xhtml

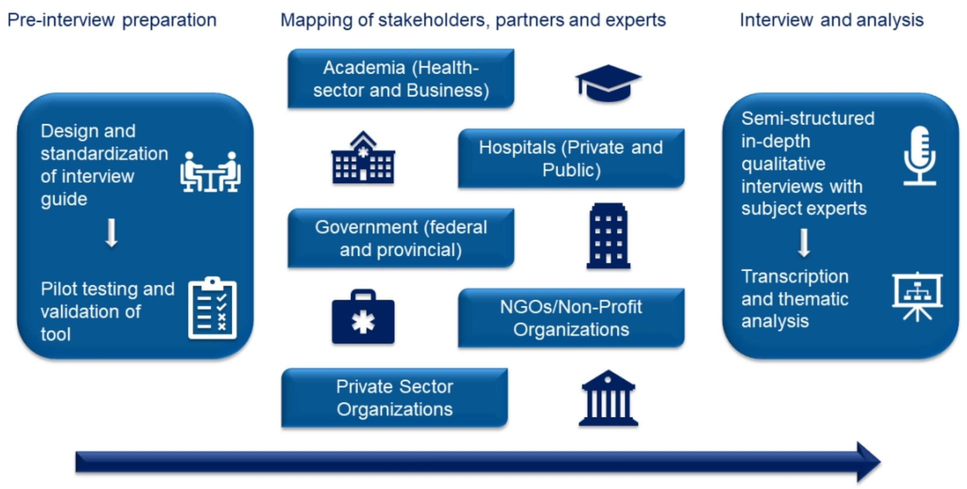

An overview of the methodological framework of the study – participant cohorts, process of interview preparation, conductance, and analysis

108x60mm (300 x 300 DPI)

Semi-structured interview guide

Section 1 : Understanding the health data landscape for Pakistan
What type of health data exists in Pakistan?
Potential prompts in case of a brief reply

- What type of data at a national/regional/global level supports your decision-making ability/research work?
- What type of health data would further support your ability to make informed decisions?
- Is health data at a Pakistan level accessible?
- Is health data at a Pakistan level of good quality? (define quality)

Section 2: Understanding the application of a gender and equity lens to data
Potential prompts in case of a brief reply (mostly guided by the interviewee's response)

- Do we know how to apply a gender/equity lens to our data (disaggregation, analysis etc)
- What population group do you not frequently see available data about?

Section 3: Understanding the organizational handle on health data and its current role
Potential prompts in case of a brief reply (mostly guided by the interviewee's response)

- What health data does your organization hold and to what level does your organization engage with the data for decision making.
- How equipped are organizations to manage the health data they hold?
- What kind of infrastructure/software does your organization have? Is it sufficient?

Section 4: Understanding perceptions around developing a health data science training program/curriculum
How effective do you think the introduction of a health data science training curriculum will be, to address barriers?
Potential prompts in case of a brief reply (mostly guided by the interviewee's response)

- What type of training in data science would be most beneficial to you and why?
- What health data science curriculum/training programs exist and are useful?
- Do you think there's an existing need for development of such a program in Pakistan? Why or why not?
- What barriers should such a training program address?
- What components should the health data science training curriculum have?

For peer review only

Consolidated criteria for reporting qualitative studies (COREQ): 32-item checklist

No. Item	Guide questions/description	Reported on Page #
**Domain 1: Research team and reflexivity**		
Personal Characteristics		
1. Inter viewer/facilitator	Which author/s conducted the interview or focus group?	7
2. Credentials	What were the researcher's credentials? E.g., PhD, MD	8
3. Occupation	What was their occupation at the time of the study?	8
4. Gender	Was the researcher male or female?	7
5. Experience and training	What experience or training did the researcher have?	8
Relationship with participants		
6. Relationship established	There was no personal relationship between interviewers	N/A
7. Participant knowledge of the interviewer	What did the participants know about the researcher? e.g., personal goals, reasons for doing the research	N/A
8. Interviewer characteristics	What characteristics were reported about the inter viewer/facilitator? e.g., Bias, assumptions, reasons and interests in the research topic	N/A

**Domain 2: study design**		
Theoretical framework		

For peer review only - http://bmjopen.bmj.com/site/about/guidelines.xhtml

9. Methodological orientation and Theory	What methodological orientation was stated to underpin the study? e.g., grounded theory, discourse analysis, ethnography, phenomenology, content analysis	9
Participant selection		
10. Sampling	How were participants selected? e.g., purposive, convenience, consecutive, snowball	8
11. Method of approach	How were participants approached? e.g., face-to-face, telephone, mail, email	8
12. Sample size	How many participants were in the study?	10
13. Non-participation	How many people refused to participate or dropped out? Reasons?	N/A
Setting		
14. Setting of data collection	Where was the data collected? e.g., home, clinic, workplace	8
15. Presence of non-participants	Was anyone else present besides the participants and researchers?	8
16. Description of sample	What are the important characteristics of the sample? e.g., demographic data, date	10
Data collection		
17. Interview guide	Were questions, prompts, guides provided by the authors? Was it pilot tested?	7
18. Repeat interviews	Were repeat inter views carried out? If yes, how many?	N/A
19. Audio/visual recording	Did the research use audio or visual recording to collect the data?	9

For peer review only

For peer review only - http://bmjopen.bmj.com/site/about/guidelines.xhtml

20. Field notes	Were field notes made during and/or after the interview or focus group?	N/A
21. Duration	What was the duration of the inter views or focus group?	8
22. Data saturation	Was data saturation discussed?	8
23. Transcripts returned	Were transcripts returned to participants for comment and/or correction?	N/A
**Domain 3: analysis and findings**		
Data analysis		
24. Number of data coders	How many data coders coded the data?	9
25. Description of the coding tree	Did authors provide a description of the coding tree?	N/A
26. Derivation of themes	Were themes identified in advance or derived from the data?	9
27. Software	What software, if applicable, was used to manage the data?	9
28. Participant checking	Did participants provide feedback on the findings?	N/A
Reporting		
29. Quotations presented	Were participant quotations presented to illustrate the themes/findings? Was each quotation identified? e.g., participant number	11-17
30. Data and findings consistent	Was there consistency between the data presented and the findings?	10-17
31. Clarity of major themes	Were major themes clearly presented in the findings?	10-17
32. Clarity of minor themes	Is there a description of diverse cases or discussion of minor themes?	N/A

For peer review only

For peer review only

For peer review only - http://bmjopen.bmj.com/site/about/guidelines.xhtml
